# The PERK-eIF2α-ATF4 Axis Is Involved in Mediating ER-Stress-Induced Ferroptosis via DDIT4-mTORC1 Inhibition and Acetaminophen-Induced Hepatotoxicity

**DOI:** 10.3390/antiox14030307

**Published:** 2025-03-03

**Authors:** Thu-Hang Thi Nghiem, Kim Anh Nguyen, Fedho Kusuma, Soyoung Park, Jeongmin Park, Yeonsoo Joe, Jaeseok Han, Hun Taeg Chung

**Affiliations:** 1 Department of Biological Sciences, University of Ulsan, Ulsan 44610, Republic of Korea; thuhang0212@ulsan.ac.kr; 2 Department of Integrated Biomedical Science, Soonchunhyang University, Cheonan 31151, Republic of Korea; nguyenkimanh@sch.ac.kr (K.A.N.); fedhokusuma@sch.ac.kr (F.K.); sypark03@sch.ac.kr (S.P.); 3 College of Korean Medicine, Daegu Haany University, Gyeongsan 38610, Republic of Korea; jpark2368@dhu.ac.kr (J.P.); joeyeonsoo@dhu.ac.kr (Y.J.); 4 Soonchunhyang Institute of Medi-Bio Science (SIMS), Soonchunhyang University, Cheonan 31151, Republic of Korea

**Keywords:** ER stress, ferroptosis, PERK, DDIT4, GPX4, unfolded protein response

## Abstract

Ferroptosis, a regulated form of cell death characterized by lipid peroxidation and iron accumulation, is increasingly recognized for its role in disease pathogenesis. The unfolded protein response (UPR) has been implicated in both endoplasmic reticulum (ER) stress and ferroptosis-mediated cell fate decisions; yet, the specific mechanism remains poorly understood. In this study, we demonstrated that ER stress induced by tunicamycin and ferroptosis triggered by erastin both activate the UPR, leading to the induction of ferroptotic cell death. This cell death was mitigated by the application of chemical chaperones and a ferroptosis inhibitor. Among the three arms of the UPR, the PERK-eIF2α-ATF4 signaling axis was identified as a crucial mediator in this process. Mechanistically, the ATF4-driven induction of DDIT4 plays a pivotal role, facilitating ferroptosis via the inhibition of the mTORC1 pathway. Furthermore, acetaminophen (APAP)-induced hepatotoxicity was investigated as a model of eIF2α-ATF4-mediated ferroptosis. Our findings reveal that the inhibition of eIF2α-ATF4 or ferroptosis protects against APAP-induced liver damage, underscoring the therapeutic potential of targeting these pathways. Overall, this study not only clarifies the intricate role of the PERK-eIF2α-ATF4 axis in ER-stress-and erastin-induced ferroptosis but also extends these findings to a clinically relevant model, providing a foundation for potential therapeutic interventions in conditions characterized by dysregulated ferroptosis and ER stress.

## 1. Introduction

Ferroptosis is a distinct mode of cell death that fundamentally differs from apoptosis and autophagy [1,2]. It is characterized by the iron-dependent peroxidation of unsaturated fatty acids in the cell membrane [3], a process that leads to extensive lipid damage, disrupting cellular integrity and, ultimately, resulting in cell death [4]. This form of cell death is regulated through multiple mechanisms, including an iron metabolism, mitochondrial function, redox homeostasis, and various signaling pathways that respond to the stresses from different cellular organelles including the mitochondria and ER [5,6].

The ER is an essential organelle in eukaryotic cells, central to protein synthesis and folding, lipid metabolism, and calcium regulation [7,8]. When its function is impaired by various factors, ER stress occurs due to the accumulation of misfolded and unfolded proteins, triggering the UPR [9,10]. The UPR in mammals comprises three signaling pathways initiated by the ER transmembrane proteins: PKR-like ER kinase (PERK) [11], inositol-requiring transmembrane kinase/endoribonuclease-1α (IRE1α) [12], and activating transcription factor 6 (ATF6) [13]. Although the UPR initially protects cells by attempting to restore homeostasis following stress, prolonged or unresolved stress can lead to apoptotic cell death through the PERK-eIF2α-ATF4-CHOP or IRE1α-ASK-JNK pathways [14,15]. Notably, although CHOP (also known as DDIT3) is well-known for its role in inducing apoptosis, our previous finding indicates that neither ATF4 nor CHOP directly targets major apoptotic genes in mouse embryonic fibroblasts (MEFs) subjected to tunicamycin (Tm)- or thapsigargin-induced ER stress [16]. Instead, they contribute to the increased production of reactive oxygen species (ROS) to induce cell death, suggesting that mechanisms other than apoptosis also might be involved in ER-stress-mediated cell death [16].

Emerging evidence underscores a complex interplay between ferroptosis and ER-stress-mediated cell death [17]. Notably, the pharmacological inhibition of the cystine–glutamate exchange can trigger both ER stress and ferroptosis through an eIF2α-ATF4-dependent upregulation of the CHAC1 gene, leading to glutathione depletion [18,19]. Furthermore, the activation of PERK has been shown to regulate the sensitivity of hepatocellular carcinoma cells to high-LET carbon ions, influencing cell fate decisions toward either apoptosis or ferroptosis [20]. Similarly, the activation of the eIF2α-ATF4 pathway is implicated in promoting ferroptosis in various cell types, including glioma cells [21], cardiomyocytes [22], and prostate cancer cells [23]. Conversely, there are indications that ER stress can negatively regulate ferroptosis, potentially as a protective mechanism against stress via the ATF4-dependent activation of HSPA5 [24,25]. Moreover, ATF4 has been observed to protect neuronal cells from death by modulating ferroptosis [26]. These observations suggest a dual role for the PERK-eIF2α-ATF4 pathways in ferroptosis, necessitating further investigation to delineate their specific roles in ferroptosis and related pathologies.

In this study, our aim was to investigate the interplay between ER stress and ferroptotic signaling, with the hypothesis that the activation of the PERK branch of the UPR under ER stress promotes ferroptosis through an ATF4-dependent mechanism. We explored this relationship both in an in vitro and in vivo animal model.

## 2. Materials and Methods

### 2.1. Animal Protocols

Eight-to-ten-week-old male C57BL/6 mice were purchased from Orient (RaonBio, Yongin, Republic of Korea). The mice were housed in an environment maintained at 25 ± 2 °C with free access to food (5053, Pico lab, Gray Summit, MO, USA) and water under a 12 h light/dark cycle with lights on from 7:00 to 19:00. All animal care and experimental procedures were conducted according to the protocols and guidelines approved by the Soonchunhyang University Animal Care and Use Committee (SCH25-0006).

The APAP-induced acute liver injury murine model was established by fasting mice overnight (17:00–09:00, 16 h) before being intraperitoneally (I.P.) injected with vehicle or 2BAct (10 mg/kg) or Ferrostatin-1 (Fer-1) (10 mg/kg) (*n* = 3–6 for each group). After 1 h of pretreatment, PBS (21-040-CV, Corning, VA, USA) or APAP (200 mg/kg) was administered via I.P. injection. Fasting had been maintained continuously post-injection for 3 or 6 h before the mice were sacrificed.

### 2.2. Hepatic Damage Assay

Activity of alanine aminotransferase (ALT) and aspartate aminotransferase (AST) in serum, as indicators of hepatic injury, were measured using the EnzyChrom ALT assay kit (EALT-100, BioAssay Systems, Hayward, CA, USA) and EnzyChrom AST assay kit (EASTR-100, Bi-oAssay Systems, Hayward, CA, USA) from BioAssay Systems under the guidance of the manufacturer’s instruction.

### 2.3. Chemicals and Reagents

Erastin (E7781, Sigma-Aldrich, St. Louis, MO, USA), SB202190 (S7067, Sigma-Aldrich, St. Louis, MO, USA), GSK2606414 (GSK) (516535, Sigma-Aldrich, St. Louis, MO, USA), Tm (T7765, MilliporeSigma, Burlington, MA, USA), MTT (M2128, Sigma-Aldrich, St. Louis, MO, USA), and ISRIB (SML0843, Sigma-Aldrich, St. Louis, MO, USA) were purchased from Sigma-Aldrich. RSL3 (S8155, Selleck Chemicals, Houston, TX, USA), Fer-1 (S7243, Selleck Chemicals, Houston, TX, USA), and Z-VAD (S7023, Selleck Chemicals, Houston, TX, USA) were purchased from Selleck Chemicals. Tauroursodeoxycholic acid (TUDCA) (T1567, TCI, Tokyo, Japan) was obtained from TCI. 4-phenylbutyric acid (4-PBA) was purchased from MedChemExpress (HY-A0281, MCE, Shanghai, China). Torin1 was from Tocris-Biotech (4247, Tocris-Biotech, Minneapolis, MN, USA). APAP was from TCI (H0190, TCI, Tokyo, Japan). 2BAct was synthesized and confirmed to be over 99% pure using 1H NMR, HPLC, and LCMS analyses by the Medicinal Chemistry Team at Daegu-Gyeongbuk Medical Innovation Foundation (Daegu, Republic of Korea).

### 2.4. Cell Culture

Alpha mouse liver 12 (AML12) cells (CRL-2254, American Type Culture Collection (ATCC), Manassas, VA, USA) were cultured in DMEM/F12 (11320033, Thermo Fisher Scientific, Waltham, MA, USA) containing 10% fetal bovine serum (FBS) (35-015-CV, Corning, NY, USA), 1% penicillin/streptomycin solution (30-002-CI, Corning, NY, USA), 1% ITS Liquid Media Supplement (I3146, Sigma-Aldrich, St. Louis, MO, USA), and 40 ng/mL dexamethasone (52-02-2, Sigma-Aldrich, St. Louis, MO, USA). The human hepatocellular carcinoma cell line HepG2 was purchased from ATCC (HB-8065, ATCC, Manassas, VA, USA) and maintained in DMEM (10-013-CV, Corning, NY, USA) supplemented with 10% FBS and 1% penicillin/streptomycin solution. *Perk*^+/+^ (wild-type) and *Perk*^−/−^ (knock-out) MEFs were cultured in DMEM medium supplemented with 10% FBS and 1% MEM nonessential amino acid solution (11140050, Gibco, NY, USA). *Atf4*^+/+^ (wild-type) and *Atf4*^−/−^ (knock-out) MEFs were cultured in DMEM medium supplemented with 10% FBS, 1% penicillin/streptomycin, 1% MEM nonessential amino acid solution, and 2.2 μL β-mercaptoethanol (21985023, Thermo Fisher Scientific, Waltham, MA, USA). *Ire1α*^+/+^ (wild-type), *Ire1α*^−/−^ (knock-out), *Atf6α*^+/+^ (wild-type), and *Atf6α*^−/−^ (knock-out) mouse hepatocytes, which were kindly provided by Dr. S. H. Back (University of Ulsan, Ulsan, Republic of Korea), were maintained in medium 199 (M4530, Sigma-Aldrich, St. Louis, MO, USA) with 10% FBS and 1% MEM nonessential amino acid solution. For cystine deprivation, the experiment was carried out by incubating cells in DMEM, high glucose, no glutamine, no methionine, no cystine (21013024, Thermo Fisher Scientific, Waltham, MA, USA) with 10% dialyzed FBS (F0392, Sigma Aldrich, St. Louis, MO, USA), that was supplemented with L-glutamine (G7513, Sigma-Aldrich, St. Louis, MO, USA) to a final concentration of 4 mM and L-methionine (4500-M, Millipore, Burlington, MA, USA) to a final concentration of 200 µM. Cells were grown in humidified incubators, at 37 °C with 5% CO_2_.

### 2.5. Real-Time Quantitative RT-PCR

Total RNA was prepared using Trizol reagent (15596026, Thermo Fisher Scientific, Waltham, MA, USA); 2 µg of total RNA was used to synthesize the first-strand cDNA with oligo (dT) primers and Moloney murine leukemia virus reverse-transcriptase (M1701, Promega, Madison, WI, USA), according to the manufacturer’s instructions. Real-time quantitative RT-PCR (qRT-PCR) was performed with TOPreal qPCR 2X PreMIX (SYBR Green with low ROX) (RT500M, Enzymnomics, Daejeon, Republic of Korea) on an ABI 7500 Fast Real-Time PCR System (Thermo Fisher Scientific, Waltham, MA, USA).

The primers used in this study are listed in Table 1.

### 2.6. Western Blot Analysis

Cell pellets and liver tissue were lysed using prepared RIPA buffer (89900, Thermo Fisher Scientific, Waltham, MA, USA) containing phosphatase and protease inhibitors (PPC1010, Sigma Aldrich, St. Louis, MO, USA), and the total protein concentration was determined by BCA protein assay reagents (23225, Pierce Biotechnology, Rockford, IL, USA) using bovine serum albumin (BSA) as the standard. Samples were boiled at 95 °C in 2x×Laemmli Sample Buffer (1610737, Bio-Rad, Hercules, CA, USA) for 5 min. Proteins were resolved by SDS-PAGE and transferred to polyvinylidene difluoride membranes (IPVH00010, Millipore, Burlington, MA, USA). The membranes were blocked with 5% nonfat milk (BD232100, BD bioscience, San Diego, CA, USA) in phosphate-buffered saline-Tween 20 (PBS-T), and then the membranes were incubated with primary antibodies as follows: p-PERK (1:1000, Cat#12814, Signalway antibody, Greenbelt, MD, USA), PERK (1:1000, Cat#5683, Cell Signaling, Danvers, MA, USA), p-eIF2α (1:1000, Cat#9721, Cell Signaling, Danvers, MA, USA), eIF2α (1:1000, Cat#9722, Cell Signaling, Danvers, MA, USA), ATF4 (1:1000, Cat#10835-1-AP, Proteintech, Rosemont, IL, USA), GRP78 (1:1000, Cat#ADI-SPA-826, Enzo Life Sciences, Farmingdale, NY, USA), DDIT3 (1:500, Cat#sc-7351, Santa Cruz, Dallas, TX, USA), p-IRE1α (1:1000, Cat#NB100-2323, Novus Biologicals, Centennial, CO, USA), total-IRE1α (1:500, Cat#sc-390960, Santa Cruz, Dallas, TX, USA), ATF6 (1:1000, #Cat24169-AP, Proteintech, Rosemont, IL, USA), XBP-1(s) (1:1000, Cat#647502, BioLegend, San Diego, CA, USA), GPX4 (1:2000, Cat#Ab125066, Abcam, Cambridge, UK), SLC7A11 (1:1000, Cat#PA1-16775, Thermo Fisher Scientific, Waltham, MA, USA), MDA (1:1000, Cat#6463, Abcam, Cambridge, UK), 4-HNE (1:1000, Cat#46545, Abcam, Cambridge, UK), CHAC1 (1:1000, Cat#15207-AP, Proteintech, Rosemont, IL, USA), DDIT4 (1:1000, Cat#10638-1-AP, Proteintech, Rosemont, IL, USA), cleaved caspase-3 (1:1000, Cat#9661, Cell signaling, Danvers, MA, USA), caspase-3 (1:1000, Cat#9662, Cell signaling, Danvers, MA, USA), p-mTOR (1:1000, Cat#2971, Cell signaling, Danvers, MA, USA), total-mTOR (1:1000, Cat#2972, Cell signaling, Danvers, MA, USA), p-4EBP1 (1:1000, Cat#9455, Cell signaling, Danvers, MA, USA), total-4EBP1 (1:1000, Cat#9452, Cell signaling, Danvers, MA, USA), p-S6 ribosomal protein (1:2000, Cat#5364, Cell signaling, Danvers, MA, USA), S6 ribosomal protein (1:2000, Cat#2317, Cell signaling, Danvers, MA, USA), and β-actin (1:2000, #Cat4967, Cell signaling, Danvers, MA, USA) as a loading control. These were incubated overnight at 4 °C. Membranes were washed with 1X PBS-T 3 times for 10 min and incubated with HRP-conjugated mouse or rabbit secondary antibodies (115-035-003, 111-035-003, Jackson ImmunoResearch Laboratories, West Grove, PA, USA). Chemiluminescence signals were read using an Azure Biosystems C300 analyzer (Azure Biosystems, Dublin, CA, USA) with an ECL substrate (1859700, Pierce Biotechnology, Rockford, IL, USA).

#### Small Interfering RNA Transfection

Small interfering RNA (siRNA) against mouse *Ddit4* (*siDdit4*) (sc-142310, Santa Cruz, Dallas, TX, USA) was purchased from Santa Cruz Biotechnology, and negative control siRNA (*scRNA*) (AM4611, Ambion, Austin, TX, USA) was purchased from Ambion. Cells were transfected using the Lipofectamine™ 2000 (11668027, Thermo Fisher Scientific, Waltham, MA, USA) in accordance with the manufacturer’s protocol. After 36 h transfection, cells were treated with indicated drug.

### 2.7. Adenovirus Infection

Adenovirus expressing GFP (Ad-GFP) or ATF4 (Ad-ATF4) were obtained from Research Animal Resource Center (Korea Institute of Science and Technology, Seoul, Republic of Korea). Cells were plated the day prior infection. Infection with adenovirus expressing either GFP or mouse ATF4 was carried at a multiplicity of infection of 1000. After 24 h infection, the cells were washed twice and treated with erastin for 24 h.

### 2.8. Cell Viability

Cell viability was detected using an MTT assay. Cells were seeded in a 96-well plate at density of 70–80% confluence. After treatment, 20 μL of MTT (5 mg/mL) in PBS solution was directly to the cell culture medium and allowed to incubate for 1 h. The medium was then removed, and solvent (isopropanol) was added to the cells and absorbance was measured at 570 nm with SpectraMax iD3 (Molecular Devices, Sunnyvale, CA, USA).

### 2.9. Measurement of Lipid ROS

Cells were seeded into 12-well plates and cultured overnight. On the next day, cells were treated with chemicals for the indicated times. Then, cells were trypsinized, washed, suspended in PBS containing 5 μM of BODIPY™ 581/591 C11 (D3861, Thermo Fisher Scientific, Waltham, MA, USA), and incubated at 37 °C for 30 min. Cells were pelleted and resuspended in PBS. Oxidation of the polyunsaturated butadienyl portion of the dye results in a shift of the fluorescence emission peak from ~590 nm to ~510 nm. To measure the lipid peroxidation, fluorescence from cells was assessed by flow cytometry (BD Biosciences, San Jose, CA, USA) using a 488 nm laser on FL1 detector. The data were analyzed using FlowJo software 10.8.0.

### 2.10. Iron Measurement

The intracellular iron concentration in cell lysates was assessed using an iron colorimetric assay kit (K390-100, Biovision, Milpitas, CA, USA) purchased from Biovision according to the manufacturer’s instructions. In this assay, ferric carrier proteins dissociate ferrous iron into solution in the presence of acid buffer. After reduction to the ferrous form (Fe^2+^), iron reacts with Ferene S to produce a stable-colored complex and give absorbance at 593 nm. Iron level in cellular extracts was quantified via comparison to a calibration curve generated using iron standard.

### 2.11. Transmission Electron Microscopy (TEM)

Cells were seeded in 6-well plates dishes at a density of 5 × 10^5^ cells/dish and cultured overnight. On the next day, cells were treated with erastin in indicated times. For TEM, cells were fixed overnight in 2.5% glutaraldehyde in 0.1 M cacodylate buffer (pH 7.3). Cells were rinsed three times with 0.1 M cacodylate buffer (pH 7.3) and postfixed with 2% osmium tetroxide for 1 h. Next, samples were dehydrated with a series of graded ethyl alcohol solutions. The samples were next embedded in EPON 812. Ultrathin sections (70–80 nm) were obtained using an ultramicrotome, co-stained with uranyl acetate and lead citrate, and examined using a transmission electron microscope (JEM-1010; JEOL, Tokyo, Japan).

### 2.12. Histology and Immunohistochemistry

Paraformaldehyde (SM-P01-100, GeneAll, Seoul, Republic of Korea)-fixed liver tissues were processed through a series of ethanol gradients and xylene, then embedded in paraffin (39601095, Leica, Nussloch, Germany). Paraffin-embedded tissue was sectioned at 5 μm using Automatic Rotary Microtome (Leica, Nussloch, Germany), then deparaffinized and rehydrated prior to downstream staining.

For H&E staining, rehydrated tissue sections were stained with hematoxylin (MA0101010, Clearview, McKinney, TX, USA) and eosin (MA0101015, Clearview, McKinney, TX, USA) to assess the severity of liver injury.

For immunohistochemistry (IHC) to detect 4-HNE, rehydrated tissue sections were incubated briefly with 3% H_2_O_2_ (081-04215, FUJIFILM Wako Chemicals, Osaka, Japan) for 10 min to block endogenous peroxidase. The sections then underwent heat-mediated antigen retrieval by immersion in sodium citrate buffer (pH 6.0) (SR2135, Biosesang, Daejeon, Republic of Korea) under 15 psi pressure for 20 min. Subsequently, the sections were cooled down to room temperature before being blocked with blocking buffer (5% Fish Serum Blocking Buffer (37527, Thermo Fisher Scientific, Waltham, MA, USA), 0.1% BSA (SM-BOV-100, Solmate, Gwangju, Republic of Korea), and 0.02% Triton X-100 (TRX506.100, Bioshop, Seoul, Republic of Korea) in PBS) for 1 h. The primary antibody 4-HNE (ab46545, Abcam, Cambridge, UK) was then incubated overnight with the sections at 4 °C. Following washing with PBS, the sections were incubated with HRP-conjugated rabbit secondary antibodies (111-035-003, Jackson ImmunoResearch Laboratories, West Grove, PA, USA) at room temperature for 2 h. Sections were developed using DAB staining kit (SK-4100, Vector Laboratories, Newark, CA, USA) before being lightly counterstained with hematoxylin. After dehydration using a series of ethanol and clearance using xylene, the obtained slides were mounted using mounting medium (SP15-100, Thermo Fisher Chemical, Waltham, MA, USA).

H&E images and IHC images were captured using a BX61 microscope (Olympus, Tokyo, Japan). The percentage of damage areas and 4-HNE positive areas were assessed using ImageJ software (version number; 1.53 k).

### 2.13. RNA Sequencing

The gene expression profiles of GSE29929, GSE227134, and GSE104601 were obtained from NCBI-GEO database. The gene expression profile of PRJNA744757 was retrieved from NCBI-SRA and analyzed to obtain raw read count data by using Galaxy website (https://usegalaxy.org/, accessed on 12 February 2024). In our RNA sequencing (RNA-seq) experiment, total RNA from 3 DMSO-treated samples and 3 erastin-treated samples was sequenced and analyzed by BGI Genomics Co., Ltd. (Hong Kong). Log2 fold change and *p*-value were calculated using DESeq2 package in R software version 4.4.1. Gene set enrichment analysis (GSEA) was performed using fgsea package in R software. The Venn diagram was created using Interactive Venn tool (https://www.interactivenn.net/, accessed on 12 February 2024). The heatmap was generated using Morpheus website (https://software.broadinstitute.org/morpheus/ accessed on 12 February 2024).

### 2.14. Statistical Analysis

Data were analyzed with Graphpad Prism 9.5.1 (GraphPad Software, San Diego, CA, USA). All values are expressed as means ± SD. Statistical analyses were performed using Student t-test or one-way ANOVA or two-way ANOVA with Tukey post hoc tests.

## 3. Results

The UPR pathways play important roles in ferroptosis signaling upon ER stress or erastin-induced ferroptosis

**Figure 1:**
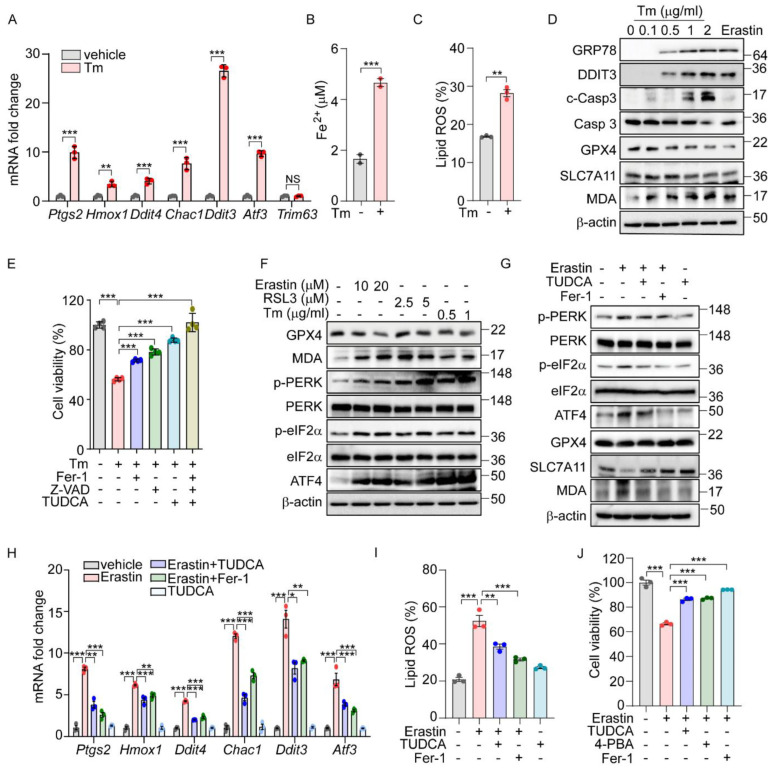
The UPR is involved in ferroptosis signaling pathway. (**A**–**C**) AML12 cells were treated with Tm (1 μg/mL) for 12 h. (**A**) Quantification of mRNA levels of indicated genes. (**B**) Intracellular ferrous iron level. (**C**) Lipid ROS was stained with BODIPY™ 581/591 C11 and measured by flow cytometry. (**D**) Protein levels of ER stress markers (GRP78 and DDIT3), apoptosis marker (cleaved caspase 3), and ferroptosis markers (GPX4, SLC7A11, and MDA) assessed by Western blot in AML12 cells after Tm treatment (0, 0.1, 0.5, 1, and 2 μg/mL) for 12 h or treatment with erastin (10 μM) for 24 h. (**E**) Cell viability in AML12 cells assessed by MTT after Tm (1 μg/mL) treatment in the presence or absence of Fer-1 (5 μM), Z-VAD (20 μM), or TUDCA (2 mM) for 12 h. (**F**) Western blot analysis of indicated proteins in AML12 cells after treatment with erastin (10 and 20 μM) for 24 h, RSL3 (2.5 and 5 μM) for 24 h, and Tm (0.5 and 1 μg/mL) for 12 h. (**G**–**J**) AML12 cells was treated with erastin (10 μM) in the presence or absence of TUDCA (1 mM), Fer-1 (5 μM), or 4-PBA (0.5 mM) for 24 h. (**G**) Western blot analysis of indicated proteins. (**H**) Quantification of mRNA levels of indicated genes. (**I**) Lipid ROS was stained with BODIPY™ 581/591 C11 and measured by flow cytometry. (**J**) Cell viability assesed by MTT. Data are mean ± SD (*n* = 3); * *p* < 0.05, ** *p* < 0.01, *** *p* < 0.001, and NS, not significant.

Previously, we demonstrated that the PERK-eIF2α-ATF4 signaling pathway triggers ROS-mediated apoptosis in response to ER stress [16]. Interestingly, our ChIP-Seq analysis did not identify the direct targeting of apoptotic genes by ATF4. Recent studies suggest that ATF4 regulates several ferroptosis-related genes, indicating that ER stress may also induce cell death through an ATF4-mediated ferroptotic pathway. To further explore this possibility, we analyzed gene expression profiles from two public datasets: GSE29929, which examines chemically induced ER stress in the liver, and GSE227134, which investigates similar stress conditions in MEFs [27,28]. Both datasets demonstrated the significant enrichment of the UPR and ferroptosis signaling pathways (Supplementary Figure S1A,B). In alignment with these data, treatment with Tm, an N-glycosylation inhibitor known to induce ER stress, significantly altered the expression of ferroptosis-associated genes such as *Ptgs2*, *Hmox1*, and *Chac1*, as well as ER-stress-related genes including *Ddit3*, and *Atf3*, but did not affect the expression of the *Trim63* gene which is not implicated in either ER stress or ferroptosis (Figure 1A). This treatment also led to a significant increase in hallmark indicators of ferroptosis, including iron level and lipid peroxidation (Figure 1B,C). Concurrently, we observed an upregulation of ER stress marker proteins such as GRP78 and DDIT3, alongside a decrease in GPX4 and SLC7A11 protein levels but an increase in Malondialdehyde (MDA) levels (Figure 1D). These observations mirror the effects seen with erastin treatment, suggesting a similar activation of ferroptosis signaling pathways. Interestingly, cell death induced by Tm was effectively mitigated by both Z-VAD (an apoptosis inhibitor) and ferrostatin-1 (Fer-1, a ferroptosis inhibitor) (Figure 1E). In contrast, cleaved caspase 3 was detected following Tm treatment but not after erastin treatment (Figure 1D), and caspase 3 cleavage was inhibited only by Z-VAD, not by Fer-1 (Supplementary Figure S1C). In addition, we observed a protective effect from TUDCA, a chemical chaperone known to relieve ER stress (Figure 1E). These findings indicate that ER stress not only triggers apoptosis but also actively induces the ferroptosis signaling pathway, leading to ferroptotic cell death.

We next explored whether the UPR is activated in chemically induced ferroptotic cell death. Treatment with erastin and RSL3, recognized inducers of ferroptosis, resulted in a dose-dependent decrease in the GPX4 level and a corresponding increase in the MDA level (Figure 1F). Additionally, both chemicals, along with Tm, led to the elevated phosphorylation of PERK and eIF2α, as well as increased ATF4 levels (Figure 1F). Notably, the enhanced phosphorylation of IRE1α and the presence of spliced forms of XBP1 and ATF6 were specifically observed following treatments with erastin and Tm, but not with RSL3 (Supplementary Figure S2A). This suggests that the activation of the IRE1α and ATF6 pathways may not be directly linked to the downstream effects of GPX4 in the ferroptotic pathway.

To further investigate the role of ER stress and the UPR in ferroptosis induced by erastin, we employed TUDCA. The treatment with TUDCA or Fer-1 effectively reversed the erastin-triggered alterations in the expression of ferroptosis-associated proteins, including GPX4, SLC7A11, and the level of MDA. Additionally, these treatments significantly reduced the elevated levels of phosphorylated PERK, eIF2α, and ATF4 induced by erastin, confirming their effectiveness in mitigating the cellular stress responses (Figure 1G). The elevated levels of ferroptosis-related genes and lipid peroxidation brought about by erastin or RSL3 were markedly reduced by Fer-1 or TUDCA (Figure 1H,I and Supplementary Figure S2C). Moreover, the reduction in cell viability observed following erastin treatment was significantly reversed with these treatments (Figure 1J). Corroborating these findings, treatment with another chemical chaperone, 4-PBA, similarly inhibited the activation of the UPR signaling pathway (Supplementary Figure S2B), reduced lipid peroxidation (Supplementary Figure S2D), and improved cell viability triggered by erastin (Figure 1J). These results strongly support that ferroptosis induced by erastin contributes to ER stress, subsequently triggering the PERK-eIF2α-ATF4 signaling pathway, which plays a crucial role in mediating ferroptotic cell death.

The PERK arm of the UPR is important for the induction of the ferroptotic pathway by erastin.

**Figure 2:**
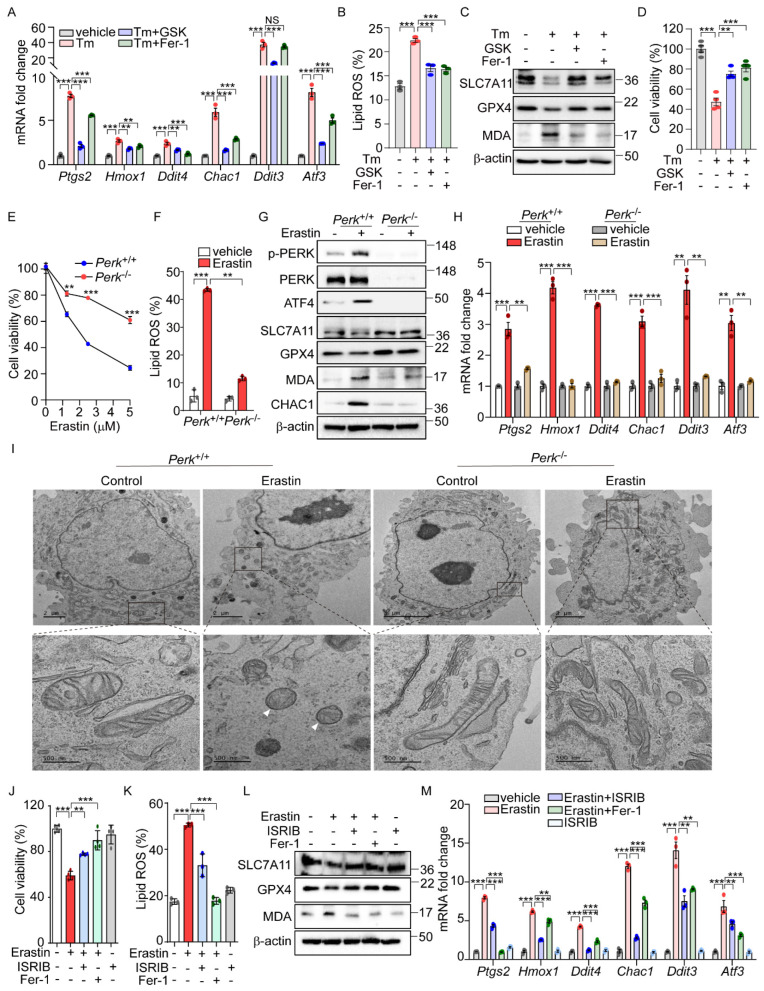
PERK and its downsteam pathway is involved in the activation of ferroptic signaling. (**A**–**D**) AML12 cells were treated with Tm (1 μg/mL) in the presence or absence of GSK2606414 (0.5 μM) or Fer-1 (5 μM) for 12 h. (**A**) Quantification of mRNA levels of indicated genes. (**B**) Lipid ROS was stained with BODIPY™ 581/591 C11 and measured by flow cytometry. (**C**) Western blot analysis of indicated proteins. (**D**) Cell viability assessed by MTT. (**E**) *Perk*^+/+^ and *Perk*^−/−^ MEFs were treated with erastin (0, 1, 2.5, and 5 μM) for 6 h, and cell viability was analyzed by MTT assay. (**F**–**I**) Erastin (2.5 μM) was treated in *Perk*^+/+^ and *Perk*^−/−^ cells for 6 h. (**F**) Lipid ROS was stained with BODIPY™ 581/591 C11 and measured by flow cytometry. (**G**) Western blot analysis of indicated proteins. (**H**) Quantification of mRNA levels of indicated genes. (**I**) Representative images of transmission electron microscopy images. White arrows indicate mitochondrial shrinkage, increased electron density, and rupture of the outer mitochondrial membrane. (**J**–**M**) AML12 cells were treated with erastin (10 μM) in the presence or absence of ISRIB (1 μM) or Fer-1 (5 μM) for 24 h. (**J**) Cell viability assessed by MTT. (**K**) Lipid ROS was stained with BODIPY™ 581/591 C11 and measured by flow cytometry. (**L**) Western blot analysis of indicated proteins. (**M**) Quantification of mRNA levels of indicated genes. Data are mean ± SD (*n* = 3); ** *p* < 0.01, *** *p* < 0.001, and NS, not significant.

Our analysis of datasets GSE29929 and GSE227134 revealed the significant enrichment of both the UPR and ferroptosis signaling pathways (Supplementary Figure S1A,B). However, in PERK knockout (KO) liver and MEFs under ER stress, the ferroptosis signaling pathway was not significantly activated, suggesting the potential role of PERK in ER-stress-mediated ferroptosis. Further emphasizing PERK’s importance, we demonstrated that GSK2606414 [29], a well-known PERK inhibitor, effectively reduced the induction of ferroptosis-related genes and lipid peroxidation by Tm treatment to levels comparable to those achieved by Fer-1 (Figure 2A,B). GSK2606414 reversed the reductions in SLC7A11 and GPX4 protein levels and the increase in MDA levels induced by Tm treatment, showing effects similar to those observed with Fer-1 treatment (Figure 2C). In addition, the diminished cell viability by Tm treatment was significantly recovered following GSK2606414 administration, similar to the recovery observed with Fer-1 treatment (Figure 2D). These findings collectively suggest that the PERK signaling pathway is involved in mediating ferroptotic cell death under ER stress conditions.

To further investigate the role of PERK signaling in erastin-induced ferroptosis, we examined ferroptotic cell death in cells with genetic deficiencies in *Ire1α*, *Atf6α*, and *Perk*. While no significant differences in cell viability were observed between *Ire1α*^+/+^ and *Ire1α*^−/−^ or between *Atf6*^+/+^ and *Atf6*^−/−^ cells (Figure S3A,B), there was a notable improvement in cell viability in *Perk*-deficient cells following erastin treatment (Figure 2E). Consistent with these findings, lipid peroxidation induced by erastin was markedly reduced in *Perk*^−/−^ compared to *Perk*^+/+^ cells (Figure 2F). Additionally, Fer-1 treatment exhibited a protective effect on cell viability and lipid peroxidation in both *Perk*^+/+^ and *Perk*^−/−^ cells (Supplementary Figure S3C,D). Not only did erastin treatment induce cell death and lipid peroxidation, but cystine deprivation also caused these effects—albeit to a lesser extent in *Perk*^−/−^ cells compared to *Perk* ^+/+^cells (Supplementary Figure S3E). In addition, Fer-1 treatment restored cell viability and reduced lipid peroxidation in both cell types (Supplementary Figure S3E). Furthermore, changes in the protein levels of SLC7A11, GPX4, CHAC1, and MDA seen in *Perk*^+/+^ cells were notably reversed in *Perk*^−/−^ cells after erastin treatment (Figure 2G). The erastin-induced upregulation of ferroptosis-related genes was also significantly diminished in *Perk*^−/−^ compared to *Perk*^+/+^ cells (Figure 2H). We further investigated the impact of inhibiting PERK activity on erastin-induced ferroptosis by using GSK2606414. As effectively as Fer-1, treatment with GSK2606414 protected cells from ferroptotic death and significantly reversed the elevated levels of *Ptgs2* mRNA and lipid peroxidation, as well as corrected the altered levels of SLC7A11, GPX4, and MDA caused by erastin (Supplementary Figure S4A–D). These results strongly suggest the important role of PERK signaling in mediating the cellular response to ER-stress-mediated as well as erastin-induced ferroptosis.

Contrary to the effects of a PERK inhibitor, treatment with the PERK activator SB202190 alone induced cell death, which was prevented by Fer-1 or Z-VAD (Supplementary Figure S4E), suggesting that PERK activation leads to cell death through both ferroptosis and apoptosis. In addition, SB202190 increased *Ptgs2* expression and lipid peroxidation, decreased GPX4 protein levels, and elevated MDA levels and CHAC1 protein levels, suggesting that PERK activation may be involved in initiating ferroptotic signaling and cell death (Supplementary Figure S4F–I).

Given that changes in mitochondrial morphology are recognized as a hallmark of ferroptosis [1], we investigated whether PERK signaling could induce morphological alterations in the mitochondria. The aberrant mitochondrial morphology in *Perk*^+/+^ cells after erastin treatment was significantly improved in *Perk*^−/−^ cells treated with erastin (Figure 2I). Moreover, cells exposed to erastin exhibited dense and deformed mitochondria, which were substantially rescued by GSK2606414 (Supplementary Figure S4J). Conversely, activating PERK with SB202190 alone induced mitochondrial morphological distortions similar to those observed in erastin-treated cells (Supplementary Figure S4J). Overall, the activation of the PERK signaling pathway may contribute to the induction of ferroptotic signaling, which is accompanied by mitochondrial dysfunction.

To further confirm the role of PERK downstream signaling in erastin-induced ferroptosis, we used the chemical modulation of the eIF2α signaling pathway. Similar to the observations in *Perk*^−/−^ cells, treatment with ISRIB, an inhibitor of eIF2α-ATF4 signaling, effectively rescued the decreased cell viability caused by erastin to a similar extent as Fer-1 treatment [30]. (Figure 2J). Additionally, both ISRIB and Fer-1 significantly reduced the levels of lipid peroxidation induced by erastin (Figure 2K). ISRIB also restored the altered expression patterns of SLC7A11, GPX4, and MDA triggered by erastin (Figure 2L), and effectively prevented the increase in ferroptosis-related gene expression induced by erastin (Figure 2M). These results provide strong evidence that the PERK-eIF2a phosphorylation signaling pathway is a key mediator of erastin-induced ferroptosis.

### 3.1. ATF4 Is a Key Gene Involved in Erastin-Induced Ferroptosis

**Figure 3:**
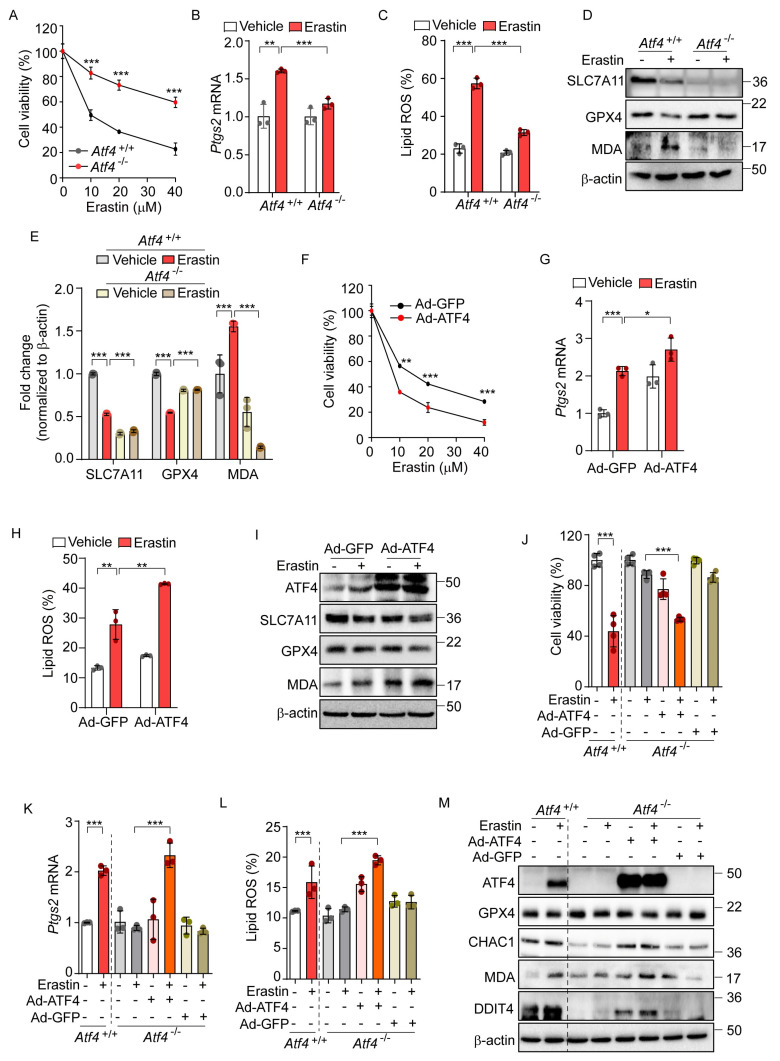
ATF4 is an important transcription factor for erastin-induced ferroptotic pathway. (**A**) Cell viability assessed by MTT in *Atf4*^+/+^ and *Atf4*^−/−^ MEFs treated with erastin (0, 10, 20, and 40 μM) for 24 h. (**B**–**E**) *Atf4*^+/+^ and *Atf4*^−/−^ MEFs were treated with erastin (10 μM) for 24 h. (**B**) Quantification of mRNA levels of Ptgs2. (**C**) Lipid ROS was stained with BODIPY™ 581/591 C11 and measured by flow cytometry. (**D**) Western blot analysis of indicated proteins. (**E**) Protein expression in panel (**D**) was quantified by image J. (**F**–**I**) Wild-type MEFs were infected with Ad-GFP or Ad-ATF4 for 24 h. (**F**) Cell viability assessed by MTT after treatment with erastin (0, 10, 20, and 40 μM) for 24 h. (**G**) Quantification of mRNA levels of Ptgs2 after erastin (10 μM) treatment for 24 h. (**H**) Lipid ROS was stained with BODIPY™ 581/591 C11 and measured by flow cytometry. (**I**) Western blot analysis of indicated proteins after erastin (10 μM) treatment for 24 h. (**J**–**M**) To rescue the expression of ATF4, Ad-GFP, or Ad-ATF4 were infected in *Atf4*^−/−^ cells in the presence or absence of erastin (10 μM) for 24 h. (**J**) Cell viability assessed by MTT. (**K**) Quantification of mRNA level of Ptgs2. (**L**) Lipid ROS was stained with BODIPY™ 581/591 C11 and measured by flow cytometry. (**M**) Western blot analysis of indicated proteins. Data are mean ± SD (*n* = 3); * *p* < 0.05, ** *p* < 0.01 and *** *p* < 0.001.

Given that ATF4 acts as a primary mediator in the PERK-eIF2α signaling pathway [31], we investigated the role of ATF4 in erastin-induced ferroptosis. We observed that *Atf4*^−/−^ cells exhibited significant resistance to the ferroptotic cell death induced by erastin, compared to *Atf4*^+/+^ cells (Figure 3A). In addition, the elevated levels of *Ptgs2* mRNA and lipid peroxidation observed in *Atf4*^+/+^ were significantly reduced in *Atf4*^−/−^ cells (Figure 3B,C). Furthermore, the erastin-induced alterations in SLC7A11, GPX4, and MDA levels observed in *Atf4*^+/+^ cells were not observed in *Atf4*^−/−^ cells (Figure 3D,E).

To further investigate the role of ATF4 in ferroptosis, we employed an adenovirus to overexpress ATF4 (Ad-ATF4), which resulted in enhanced cell death as well as elevated levels of *Ptgs2* expression and lipid peroxidation relative to a control virus expressing GFP (Ad-GFP) upon erastin treatment (Figure 3F–H). Ad-ATF4 significantly altered the expression of ferroptosis-related proteins and MDA levels upon erastin treatment compared to cells infected with Ad-GFP (Figure 3I). In subsequent experiments, we reintroduced ATF4 into *Atf4*^−/−^ cells, which had previously shown resistance to erastin-induced ferroptotic cell death. Unlike their counterparts infected with Ad-GFP, *Atf4*^−/−^ cells with Ad-ATF4 showed a markedly increased sensitivity to ferroptosis, comparable to that of *Atf4*^+/+^ cells (Figure 3J). These cells also demonstrated enhanced responsiveness to the erastin-triggered ferroptotic pathway, exhibiting increased *Ptgs2* mRNA levels, elevated lipid peroxidation, and altered expressions of GPX4, CHAC1, and DDIT4 proteins and the MDA level in response to erastin treatment, similarly to *Atf4*^+/+^ cells (Figure 3K–M). These results suggest that ATF4 is involved in the activation of ferroptosis signaling and may potentially contribute to ferroptosis-mediated cell death. 

### 3.2. ATF4-Induced Activation of DDIT4 Suppresses the mTOR Pathway and Triggers Ferroptosis

**Figure 4:**
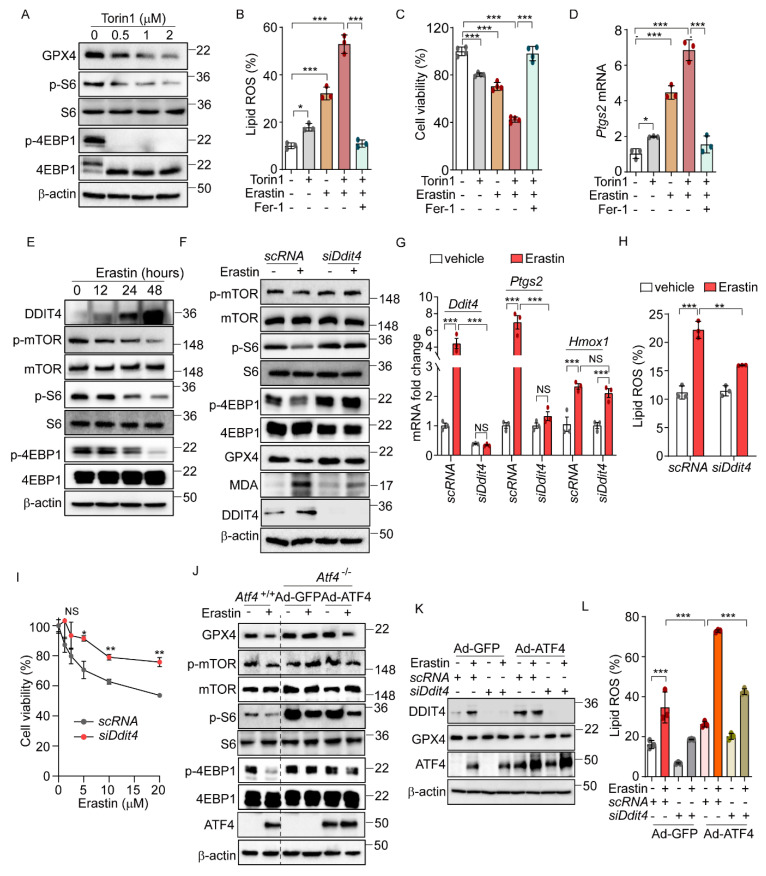
ATF4-induced DDIT4 and subsequent mTORC1 inhibition trigger ferroptotic signaling. (**A**) Western blot analysis of indicated proteins in AML12 cells after Torin1 treatment (0, 0.5, 1, and 2 μM) for 24 h. (**B**–**D**) AML12 cells were treated with erastin (5 μM) or Torin1 (0.5 μM) in the presence or absence of Fer-1 (10 μM) for 16 h. (**B**) Lipid ROS was stained with BODIPY™ 581/591 C11 and measured by flow cytometry. (**C**) Cell viability assessed by MTT. (**D**) Quantification of mRNA level of *Ptgs2*. (**E**) Western blot analysis of indicated proteins in AML12 after treatment with erastin (10 μM) at indicated times. (**F**–**I**) For knockdown *Ddit4*, AML12 cells were transfected with control siRNA (*scRNA*) or *siDdit4* for 36 h. (**F**) Western blot analysis of indicated proteins after erastin (10 μM) treatment for 24 h. (**G**) Quantification of mRNA level of indicated genes. (**H**) Lipid ROS was stained with BODIPY™ 581/591 C11 and measured by flow cytometry. (**I**) Cell viability assessed by MTT assay after erastin treatment (0, 1.25, 2.5, 5, 10, and 20 μM) for 24 h. (**J**) *Atf4*^−/−^ cells were infected with Ad-GFP or Ad-ATF4 and then treated with erastin (10 μM) for 24 h. Western blot analysis of indicated proteins. (**K**–**L**) Ad-GFP and Ad-ATF4 were infected in *scRNA* or *siDdit4*-transfected AML12 cells followed by erastin (10 μM) treatment for 24 h. (**K**) Western blot analysis of indicated proteins. (**L**) Lipid ROS was stained with BODIPY™ 581/591 C11 and measured by flow cytometry. Data are mean ± SD (*n* = 3); * *p* < 0.05, ** *p* < 0.01, *** *p* < 0.001, and NS, not significant.

Previous research has shown that the sustained activation of the mTOR pathway can inhibit ferroptosis through the promotion of GPX4 protein synthesis [31,32]. Considering the known crosstalk between the eIF2α-ATF4 and the mTOR signaling pathway [31], we hypothesized that eIF2α-ATF4 might mediate ferroptosis through the modulation of mTOR signaling. To explore this hypothesis, we first assessed the relationship between the mTOR pathway and ferroptosis using the mTOR inhibitor, Torin1. Consistent with expectations, Torin1 treatment significantly diminished mTOR signaling, as evidenced by the reduced levels of phosphorylated S6 and 4EBP1, and simultaneously activated ferroptosis, indicated by the decreased GPX4 protein level (Figure 4A). The co-administration of Torin1 and erastin resulted in increased lipid peroxidation, cell death, and upregulation of the *Ptgs2* mRNA level, showing enhanced effects compared to the treatment with erastin alone (Figure 4B–D). These enhancements were significantly mitigated by Fer-1. Similarly, the combination of Torin1 and RSL3 led to increased lipid peroxidation and cell death compared to RSL3 alone (Supplementary Figure S5A,B). In addition, Z-VAD and Fer-1 treatment improved cell survival caused by Torin1 treatment (Supplementary Figure S5C). Interestingly, Torin1 treatment alone exhibited only marginal effects on lipid peroxidation, cell viability, and *Ptgs2* gene expression levels, suggesting that mTOR inhibition by itself has a limited impact on ferroptosis. Nonetheless, these findings imply that the inhibition of the mTOR pathway plays a role in the ferroptotic signaling cascade and may potentially enhance its activation.

Next, we investigated the crosstalk between eIF2α-ATF4-mediated ferroptosis and mTOR signaling. Previous research indicated that DDIT4 inhibits mTOR signaling [33,34]. Given that DDIT4 is a target of ATF4 [16], we hypothesized that the ATF4-mediated upregulation of DDIT4 could suppress mTOR signaling, thereby enhancing the ferroptotic pathway induced by erastin. Erastin treatment induced a time-dependent increase in DDIT4 expression, accompanied by decreased levels of phosphorylated mTOR, S6, and 4EBP1 (Figure 4E). Notably, these erastin-induced changes in mTOR-pathway-related proteins were not observed in *Atf4*^−/−^ cells, and treatment with Fer-1 did not affect these proteins in *Atf4*^−/−^ cells (Supplementary Figure S5D). To further explore the role of DDIT4 in the mTOR inhibition induced by erastin, we used *siDdit4* to silence its expression. This intervention effectively restored the reduced phosphorylation levels of mTOR, S6, and 4EBP1, as well as normalized the altered levels of GPX4 and MDA that had been changed by erastin (Figure 4F). Additionally, silencing *Ddit4* reversed the elevated expression of ferroptosis-related genes and reduced the increased lipid peroxidation level caused by erastin (Figure 4G,H). Moreover, the decrease in cell viability induced by erastin was significantly improved upon *Ddit4* silencing (Figure 4I). Interestingly, *Ddit4* silencing significantly, though not completely, restores the lipid peroxidation and cell viability changes induced by erastin treatment, suggesting that, while DDIT4 plays an important role in erastin-mediated ferroptosis, additional signaling pathways may also be involved.

Further investigation into the relationship between mTOR signaling and ATF4-mediated ferroptosis revealed that erastin treatment failed to alter the levels of GPX4 or the phosphorylation status of mTOR, S6, and 4EBP1 in *Atf4*^−/−^ cells infected with Ad-GFP (Figure 4J). However, restoring the ATF4 level via Ad-ATF4 infection led to decreased levels of GPX4 and phosphorylated mTOR, S6, and 4EBP1 by erastin, suggesting that ATF4 mediates the erastin-induced mTOR inhibition and the subsequent activation of ferroptosis signaling (Figure 4J). In line with these findings, silencing *Ddit4* inhibited the erastin-induced reduction in GPX4, and ATF4 overexpression did not reverse this effect (Figure 4K). Additionally, the increased lipid peroxidation triggered by erastin and Ad-ATF4 was significantly reduced by *Ddit4* silencing (Figure 4L). These results suggest the role of the ATF4-induced DDIT4 expression in reducing mTOR signaling and sensitizing cells to ferroptosis upon erastin treatment. Interestingly, we found that *Gpx4* mRNA levels were increased in *Atf4*^−/−^, *siDdit4*, and *Perk*^−/−^ cells compared to their respective control cells (Supplementary Figure S5E–G). This observation suggests that the enhanced resistance of these cells to erastin-induced ferroptosis may be mediated not only by the inhibition of mTOR signaling but also by a PERK/ATF4/DDIT4-dependent increase in *Gpx4* mRNA levels.

### 3.3. eIF2α-ATF4 Mediates Acetaminophen-Induced Ferroptosis in the Hepatocytes

**Figure 5:**
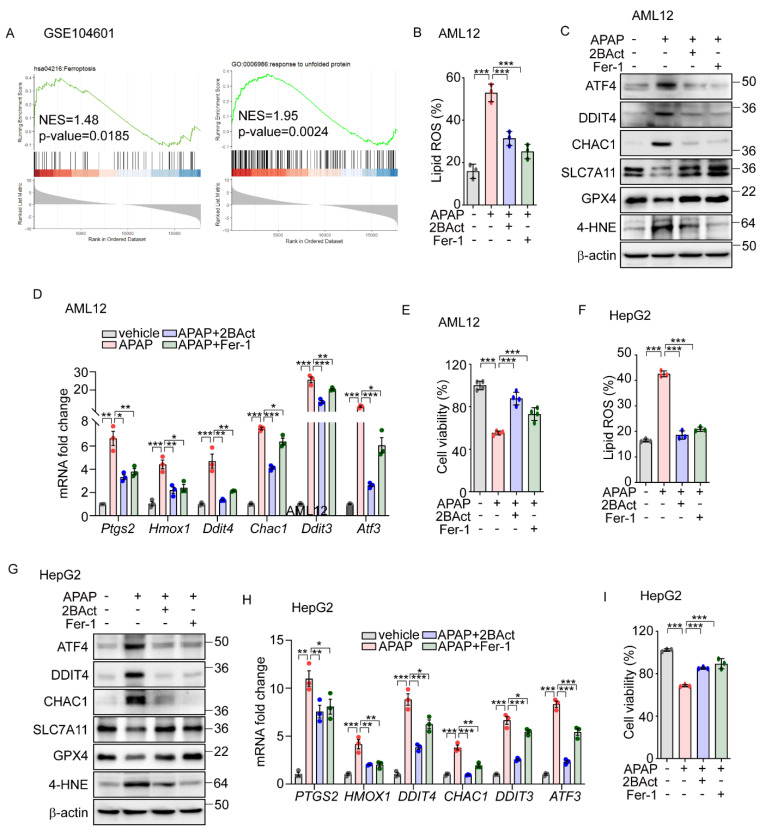
Acentaminophen-induced ferroptosis is attenuated by inhibition of ISR in murine and human hepatocytes. (**A**) GSEA plot of transcriptomes from human liver organoids treated with APAP (10 mM) for 24 h. (**B**–**E**) APAP (20 mM) was treated for 12 h in the presence or absence of 2BAct (5 μM) or Fer-1 (5 μM) in AML12 cells. (**B**) Lipid ROS was stained with BODIPY™ 581/591 C11 and measured by flow cytometry. (**C**) Western blot analysis of indicated proteins. (**D**) Quantification of mRNA level of indicated genes. (**E**) Cell viability assessed by MTT. (**F**–**I**) APAP (20 mM) was treated for 24 h in the presence or absence of 2BAct (5 μM) or Fer-1 (5 μM) in HepG2 cells. (**F**) Lipid ROS was stained with BODIPY™ 581/591 C11 and measured by flow cytometry. (**G**) Western blot analysis of indicated proteins. (**H**) Quantification of mRNA level of indicated genes. (**I**) Cell viability assessed by MTT. Data are mean ± SD (*n* = 3); * *p* < 0.05, ** *p* < 0.01 and *** *p* < 0.001.

To investigate the physiological impacts of eIF2α-ATF4 regulation on ferroptosis, we employed a model of APAP-induced liver injury. APAP is commonly used for pain and fever relief and is safe within therapeutic doses; however, at excessive doses, it can cause severe hepatic toxicity and even acute liver failure [35]. Recent research suggests that an overdose of APAP can initiate ferroptosis, leading to significant liver damage [36,37]. To explore the role of eIF2α-ATF4 in APAP-induced ferroptosis further, we first analyzed data from GSE104601 [38], which evaluates the effects of APAP on human primary hepatocytes. This analysis revealed the significant enrichment of ferroptosis and UPR signaling pathways in hepatocytes following APAP exposure (Figure 5A). Corroborating these findings, we observed increased lipid peroxidation (Figure 5B) and altered protein levels of GPX4, CHAC1, SLC7A11, and DDIT4, as well as elevated levels of 4-HNE (Figure 5C) and the upregulation of ferroptosis-related genes (Figure 5D) in the murine hepatocyte cell line AML12 following APAP treatment. These effects were significantly mitigated by the eIF2α-ATF4 inhibitor 2BAct [39], with an efficacy comparable to that of Fer-1 (Figure 5B–D). Furthermore, the reduction in cell viability induced by APAP was significantly reversed with treatments of 2BAct and Fer-1 (Figure 5E). These results suggest that APAP-induced damage involves both eIF2α-ATF4 and ferroptosis activation.

Building on data analysis from human primary hepatocytes (Figure 5A), we extended our investigation to determine whether APAP-induced eIF2α-ATF4 signaling and ferroptosis also impact human hepatocyte function. In human-hepatoma-cell-line HepG2 cells, APAP treatment significantly elevated the lipid peroxidation level which was significantly decreased by Fer-1 and 2BAct (Figure 5F). Similarly, these interventions normalized the altered protein levels of DDIT4, CHAC1, SLC7A11, and GPX4, and the amount of 4-HNE (Figure 5G). Additionally, the expression levels of ferroptosis-related genes were effectively restored by these treatments (Figure 5H). Furthermore, reductions in cell viability induced by APAP were significantly reversed by treatments with Fer-1 and 2BAct (Figure 5I). These results robustly suggest the crucial role of ferroptosis in APAP-induced hepatocyte dysfunction and highlight the mediating role of eIF2α-ATF4 signaling in this pathological process.

### 3.4. Inhibition of eIF2α-ATF4 Protects the Liver from Acetaminophen-Induced Ferroptotic Damage

**Figure 6:**
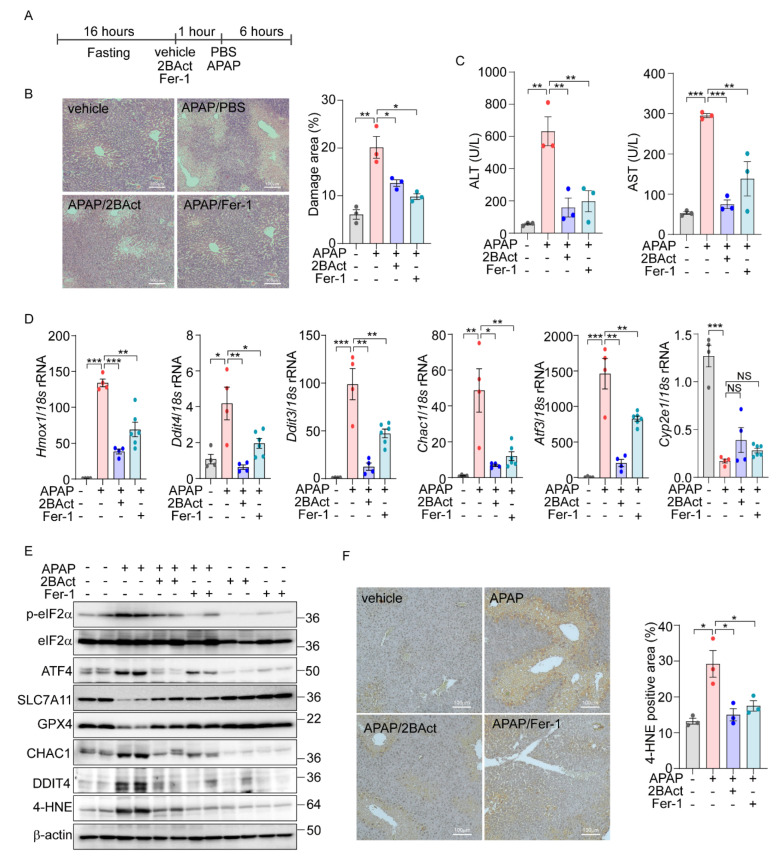
Acetaminophen-induced liver damage is mediated by eIF2a-ATF4-driven ferroptosis. (**A**–**F**) 16 h-fasted-C57BL6/J mice (8–10 weeks) were intraperitoneally injected with vehicle, 2BAct (10 mg/kg), or Fer-1 (10 mg/kg) 1 h prior to PBS or APAP (200 mg/kg) injection. (**A**) Schematic diagram of experimental design. (**B**) Representative image of liver sections stained with Hematoxylin and Eosin (H&E). The percentage of damage areas were quantified by ImageJ software. (**C**) Serum levels of AST and ALT. (**D**) Quantification of mRNA level of indicated genes. (**E**) Western blot analysis of indicated proteins. (**F**) Representative image of liver section immunohistochemically stained against 4-HNE. The percentage of 4-HNE positive areas were quantified by ImageJ software. Data are mean ± SD (*n* = 3–6); * *p* < 0.05, ** *p* < 0.01 and *** *p* < 0.001.

Next, we sought to translate our in vitro findings on eIF2α-ATF4-mediated ferroptosis into an in vivo context. Utilizing public datasets (PRJNA744757) from C57BL/6 wild-type mice treated with APAP (300 mg/kg) [40] and our own RNA-Seq data from MEFs treated with erastin, we conducted Gene Set Enrichment Analysis (GSEA). Our analysis revealed a significant upregulation of the KEGG ferroptosis pathway (mmu04216) in both APAP- and Erastin-treated samples (Supplementary Figure S6A). Additionally, the GO biological process associated with the response to unfolded protein showed significant upregulation, mirroring the cellular stress responses observed in vitro. Next, we overlapped differentially expressed genes from the in vivo datasets with ATF4 target genes identified previously [16]. This analysis revealed 86 overlapping genes, 20 of which were directly regulated by ATF4 (Supplementary Figure S6B). The expression patterns of these ATF4-regulated genes were visualized in heatmaps, providing a direct link between our in vitro discoveries and their physiological manifestations in vivo. Subsequently, we examined the specific induction of eIF2α-ATF4 signaling and ferroptosis in the liver after APAP administration. There was a notable upregulation of eIF2α-ATF4 and ferroptosis marker genes at 3 and 6 h post-injection, indicating active eIF2α-ATF4 and ferroptosis signaling pathways (Supplementary Figure S6C,D). To determine if inhibiting these pathways could mitigate APAP-induced liver damage, mice were pretreated with the eIF2α-ATF4 inhibitor 2BAct and the ferroptosis inhibitor Fer-1 (Figure 6A). Consistent with previous studies, APAP treatment led to increased necrosis in the liver, which was significantly mitigated by Fer-1. Similarly, 2BAct treatment effectively reduced the extent of necrotic areas caused by APAP (Figure 6B). In addition, the elevated ALT and AST levels induced by APAP were significantly decreased with these treatments (Figure 6C).

Further, we assessed the impact of Fer-1 and 2BAct on the gene expression levels associated with ferroptosis and eIF2α-ATF4 signaling pathway. Treatment with these inhibitors significantly attenuated the APAP-induced upregulation of ferroptosis-related and eIF2α-ATF4-specific genes (Figure 6D). Notably, the expression of *Cyp2e1*, essential for the metabolic activation of APAP, remained unchanged, indicating that the protective mechanism of the inhibitors does not alter APAP metabolism.

Protein expression analyses also supported these findings, with significant changes in the levels of ferroptosis markers such as SLC7A11, GPX4, CHAC1, and DDIT4 observed following APAP exposure. These alterations were effectively reversed by treatment with 2BAct and Fer-1 (Figure 6E and Supplementary Figure S6E). Moreover, the concentration of 4-HNE was significantly elevated in APAP-treated livers but was markedly reduced by the 2BAct and Fer-1 treatment (Figure 6E and Supplementary Figure S6E). The localization of the 4-HNE expression to zone 3 of the liver, which is most susceptible to APAP damage, was also significantly diminished following inhibitor treatment (Figure 6F). These comprehensive findings strongly support the conclusion that APAP-induced liver damage is mediated through eIF2α-ATF4-induced ferroptosis.

## 4. Discussion

Ferroptosis is a specific type of cell death that is characterized by the accumulation of lipid peroxides and cellular iron [41]. Although a relationship between ferroptosis and ER stress is recognized, the specifics of their interaction have yet to be fully delineated. In our study, we established that the PERK-eIF2α-ATF4 signaling pathway plays an important role in mediating both ER-stress- and erastin-induced ferroptosis. Our findings demonstrated that both the genetic and pharmacological inhibition of this pathway significantly alleviated ER-stress- and erastin-induced ferroptosis. Conversely, the activation of this pathway markedly enhanced ferroptotic cell death. Central to this pathway, ATF4 emerged as a key regulator, orchestrating erastin-induced ferroptosis by suppressing mTORC1 signaling via DDIT4 induction. Additionally, our findings revealed that APAP also triggers hepatocyte and liver damage through eIF2α-ATF4-mediated ferroptosis. This expands the relevance of eIF2α-ATF4 and ferroptosis beyond the context of experimental inducers like erastin to clinically relevant scenarios involving common medications, underscoring the potential for targeted therapies that modulate these pathways to mitigate drug-induced liver injuries.

Since the discovery of ferroptosis in 2012 [41], significant progress has been made in elucidating its underlying mechanisms, which include excessive intracellular iron accumulation, glutathione (GSH) depletion, glutathione peroxidase 4 (GPX4) inactivation, and increased lipid peroxidation. The role of the eIF2α-ATF4 signaling pathway in ferroptosis has been explored; yet, their exact contributions remain contentious. Several studies have shown that ATF4 may drive ferroptosis. For instance, ATF4 was necessary for ferroptosis induced by cystine starvation, erastin, and artesunate [18,34,42,43,44]. Moreover, eIF2α-ATF4 activation has been implicated in conditions such as diabetic myocardial ischemia/reperfusion injury and traumatic brain injury, suggesting a broader role for ATF4 in stress-related ferroptotic signaling [45,46]. Conversely, other research indicates that ATF4 can confer protection against ferroptosis. The activation of ATF4 by YAP/TAZ has been shown to shield hepatocellular carcinoma cells from sorafenib-induced ferroptosis [47]. Similarly, ATF4 knockdown increased the susceptibility to ferroptosis in glioma cells treated with erastin and sorafenib [26]. Additional studies have demonstrated that the ATF4-induced expression of HSPA5, also known as BiP, can prevent GPX4 degradation and protect against ferroptosis in pancreatic ductal adenocarcinoma and glioma cells [24,25,26]. A recent study also highlighted ATF4’s protective role in preventing ferroptosis in cardiomyopathy caused by a Cox10 deficiency [48]. Given these conflicting roles, it is crucial that we clarify whether eIF2α-ATF4 activation exerts a protective or detrimental effect on ferroptosis. In this study, we demonstrate that the PERK-eIF2α-ATF4 signaling pathway plays a critical role in both erastin-induced ferroptosis as well as APAP-induced hepatotoxicity. While our findings underscore the role of the eIF2α-ATF4 signaling pathway in mediating ferroptosis, the potential protective effects of eIF2α-ATF4 against ferroptosis cannot be disregarded. This apparent discrepancy may stem from the dual nature of stress responses, which might initially protect cells from adverse effects but could lead to cell death under severe and prolonged stress conditions. Further research is necessary in order to resolve these ambiguities and fully understand the intricate dynamics of eIF2α-ATF4 signaling in ferroptosis.

Cell death is an essential physiological process that plays a critical role in normal development, tissue homeostasis, and the elimination of damaged or unwanted cells [2]. There are several forms of cell death, including apoptosis, necrosis, autophagy-dependent cell death, and, more recently discovered, ferroptosis [49]. It is established that prolonged and severe ER stress can lead to cell death through the apoptotic pathway. While the IRE1α/XBP1 signaling pathway has been shown to induce apoptosis via TRAF2-ASK1 activation or the RIDD pathway [50,51], many studies have highlighted the significance of the ATF4-CHOP signaling pathway in ER-stress-mediated cell death [52,53,54]. As a transcription factor, CHOP is known to activate several apoptotic genes, including PUMA [55], Bim [56], and DR5 [57]. Additionally, ATF4 is also implicated in ER-stress-mediated apoptosis by promoting the transactivation of CHOP. Interestingly, we observed that ChIP-seq analysis for ATF4 and CHOP did not identify the major apoptotic genes but identified those genes involved in protein synthesis and some genes related to ferroptosis [16]. In that study, we found that sustained ATF4 levels increased the translation rate, leading to energy consumption and increased ROS levels, which caused apoptotic cell death. Interestingly, we also identified various ferroptosis-related genes as targets of ATF4 [16]. In line with those observations, in this study, we found that ER-stress-induced cell death was prevented by ferrostatin-1 or Z-VAD, suggesting that the induction of ATF4 could also activate the ferroptotic as well as apoptotic signaling pathway leading to cell death. Therefore, it is likely that the activation of ATF4 by ER stress can lead to cell death and either ROS- or CHOP-dependent apoptosis or DDIT4-dependent ferroptosis. However, we cannot rule out the possibility that ROS generated by PERK-eIF2α-ATF4 activation may also contribute to the activation of additional cell death pathways beyond ferroptosis and apoptosis, warranting further investigation. Furthermore, it is plausible that PERK-eIF2α-ATF4 activation could influence iron-mediated ferroptosis; however, this was not explored in the current study. Given its significance, we plan to research this in future studies.

Although we showed that the activation of eIF2α-ATF4 by PERK is involved in erastin-induced ferroptotic cell death, we cannot exclude the role of other eIF2α kinases in the ferroptotic signaling pathway induction. In fact, there are some reports that GCN2, HRI, and PKR may be involved in the stress-induced ferroptosis [16,25,34,58]; however, the exact mechanisms for how these kinases affect ferroptosis are not known. In this study, we showed that the activation of PERK by erastin caused mitochondrial morphological changes but still do not know how PERK activation induced mitochondrial morphological changes. Interestingly, PERK activation was shown to stimulate the cristae formation upon several stimuli [59,60,61]. In contrast, in line with our observations, there is another report that intermittent hypoxia in neuronal cells caused a mitochondrial cristae defect which was restored by the treatment of the PERK inhibitor [62]. Therefore, it is necessary that we elucidate the exact role of PERK in ferroptosis-related mitochondrial morphological changes.

Since its discovery, ferroptosis has been implicated in a range of physiological roles including tumor suppression, immunity, and protection against ischemic organ damage [4]. Our study extends this focus to drug-induced liver injury, specifically exploring how APAP induces hepatotoxicity through eIF2α-ATF4-mediated ferroptosis. APAP overdose is a leading cause of drug-induced liver failure in the United States, though the precise mechanisms of cell death have remained somewhat elusive [35]. Our findings reveal that APAP triggers an induction of the eIF2α-ATF4 signaling pathway and promotes ferroptosis. The link between eIF2α-ATF4 and ferroptosis was substantiated by our observations that inhibiting eIF2α-ATF4 signaling could attenuate ferroptosis activation and mitigate subsequent liver damage. Importantly, treatments targeting eIF2α-ATF4 and ferroptosis did not alter the *Cyp2e1* expression, indicating that these interventions do not affect APAP metabolism or the generation of NAPQI—the highly reactive metabolite primarily responsible for APAP-induced hepatotoxicity [63]. NAPQI is known to form protein adducts and induce mitochondrial oxidative stress and dysfunction, which, in turn, might activate the eIF2α-ATF4 signaling pathway and promote ferroptosis. These insights position the eIF2α-ATF4 signaling pathway as a potential therapeutic target for preventing liver injury from APAP overdoses. The discovery will open new avenues for using eIF2α-ATF4 inhibitors to treat or prevent liver damage, especially from drug-induced causes. However, further research is necessary in order to understand the role of the eIF2α-ATF4 signaling pathway across various models of liver damage and to determine the long-term safety of eIF2α-ATF4-targeted therapies in clinical settings.

In conclusion, our study elucidates the molecular mechanisms underlying the induction of ferroptosis by erastin, and the role of the UPR in this process, and extends these findings to a clinically relevant model of drug-induced liver injury using APAP. We demonstrate that a substantial portion of erastin-induced ferroptosis is mediated through the PERK-eIF2α-ATF4 signaling pathway and that similar pathways are activated by APAP to induce hepatotoxicity. Importantly, inhibiting the eIF2α-ATF4 signaling pathway can protect cells from ferroptotic death induced by both erastin and APAP. These insights not only advance our understanding of ferroptosis but also highlight the therapeutic potential of targeting components of the eIF2α-ATF4 signaling pathway to treat or prevent diseases associated with abnormal ferroptosis, such as drug-induced liver injury. Further research is required in order to explore how the ER-stress-induced eIF2α-ATF4 signaling pathway interacts with ferroptotic signaling pathways and to identify potential therapeutic targets for diseases characterized by these complex cellular dynamics.

## Figures and Tables

**Table 1:** List of primers for qRT-PCR analysis of gene expression. F—forward, R—reverse, bs—bases.

	Accession	Gene Name	Primer	Sequence 5′-3′	No. bs
Mouse	NM_008084.4	*Gapdh*—glyceraldehyde-3-phosphate dehydrogenase	Forward	CGGCCTCACCCCATTTG	17
			Reverse	GGGAAGCCCATCACCATCT	19
	NR_003278.3	*18s rRNA*—18S ribosomal RNA	Forward	CGCTTCCTTACCTGGTTGAT	20
			Reverse	GAGCGACCAAAGGAACCATA	20
	NM_001039048.2	*Trim63*—*Tripartite motif-containing 63*	Forward	GTGTGAGGTGCCTACTTGCTC	21
			Reverse	GCTCAGTCTTCTGTCCTTGGA	21
	NM_011198.5	*Ptgs2*—prostaglandin-endoperoxide synthase 2	Forward	TTCCAATCCATGTCAAAACCGT	22
			Reverse	AGTCCGGGTACAGTCACACTT	21
	NM_010442.2	*Hmox1*—heme oxygenase 1	Forward	AGGTGTCCAGGGAAGGCTTT	20
			Reverse	TAATGCCTTCCCTGGACACC	20
	NM_007837.5	*Ddit3*—*DNA-damage inducible transcript 3*	Forward	CTGCCTTTCACCTTGGAGAC	20
			Reverse	CGTTTCCTGGGGATGAGATA	20
	NM_029083.2	*Ddit4*—DNA-damage-inducible transcript 4	Forward	TGGTGCCCACCTTTCAGTTG	20
			Reverse	GTCAGGGACTGGCTGTAACC	20
	NM_007498.3	*Atf3*—activating transcription factor 3	Forward	CTGGAGATGTCAGTCACCAAGTCTGAG	27
			Reverse	CTCCAGTTTCTCTGACTCTTTCTGCAGGCAC	31
	NM_026929.4	*Chac1*—*ChaC glutathione specific gamma-glutamylcyclo transferase 1*	Forward	ACAAGATGAGCACCTGGAAG	20
			Reverse	GTACTGCATACTGATGTCCACC	22
	NM_021282.3	*Cyp2e1*—*cytochrome P450, family 2, subfamily e, polypeptide 1*	Forward	CAGGAGTACAAGAACAAGGGGAT	23
			Reverse	TTTGGATGCGGGCCTCATTA	20
	NM_001356512.1	*Hspd1*—*heat shock protein 1 (chaperonin)*	Forward	AGTGTTCAGTCCATTGTCCC	20
			Reverse	TGACTGCCACAACCTGAAG	19
	NM_008303.4	*Hspe1*—*heat shock protein 1 (chaperonin 10)*	Forward	GCGAAGGCGAGAGTCATG	18
			Reverse	TGCTTGCAACACTTTTCCTTG	21
Human	NM_001289746.2	GAPDH—glyceraldehyde-3-phosphate dehydrogenase	Forward	CAATGACCCCTTCATTGACCTC	22
			Reverse	AGCATCGCCCCACTTGATT	19
	NM_000963.4	PTGS2—prostaglandin-endoperoxide synthase 2	Forward	CTGGCGCTCAGCCATACAG	19
			Reverse	CGCACTTATACTGGTCAAATCCC	23
	NM_002133.3	HMOX1—heme oxygenase 1	Forward	CAGGAGCTGCTGACCCATGA	20
			Reverse	AGCAACTGTCGCCACCAGAA	20
	NM_004083.6	DDIT3—DNA damage inducible transcript 3	Forward	CAGAACCAGCAGAGGTCACA	20
			Reverse	AGCTGTGCCACTTTCCTTTC	20
	NM_019058.4	DDIT4—DNA-damage-inducible transcript 4	Forward	GAACTCCCACCCCAGATCGG	20
			Reverse	CCACTGTTGCTGCTGTCCAG	20
	NM_001206484.3	ATF3—activating transcription factor 3	Forward	GGAGCCTGGAGCAAAATGAT	20
			Reverse	AGGGCGTCAGGTTAGCAAAA	20
	NM_024111.6	CHAC1—ChaC glutathione specific gamma-glutamylcyclo transferase 1	Forward	TGGTGACGCTCCTTGAAGATC	21
			Reverse	GCACTGCCTCTCGCACATT	19

## Data Availability

The data are available in the article itself and its Supplementary Materials.

## Supplementary Materials

The following supporting information can be downloaded at: https://www.mdpi.com/article/10.3390/antiox14030307/s1.
