# Supplementary material for: The PERK-eIF2α-ATF4 Axis Is Involved in Mediating ER-Stress-Induced Ferroptosis via DDIT4-mTORC1 Inhibition and Acetaminophen-Induced Hepatotoxicity
Source: Antioxidants (Basel). 2025 Mar 3;14(3):307. doi: 10.3390/antiox14030307 (PMC11939615; doi:10.3390/antiox14030307)
Supplement: Supplementary file 1 [file antioxidants-14-00307-s001.zip › antioxidants-3386325-supplementary.pdf]

Supplementary Information

The PERK-eIF2α-ATF4 Axis Is Involved in Mediating ER-Stress-Induced Ferroptosis via DDIT4-mTORC1 Inhibition and Acetaminophen-Induced Hepatotoxicity

Thu-Hang Thi Nghiem [1,†], Kim Anh Nguyen [2,†], Fedho Kusuma [2], Soyoung Park [2], Jeongmin Park [3], Yeonsoo Joe [3], Jaeseok Han [2,4,‡] and Hun Taeg Chung [3,‡]

[1] Department of Biological Sciences, University of Ulsan, Ulsan 44610, Republic of Korea
[2] Department of Integrated Biomedical Science, Soonchunhyang University, Cheonan 31151, Republic of Korea
[3] College of Korean Medicine, Daegu Haany University, Gyeongsan 38610, Republic of Korea
[4] Soonchunhyang Institute of Medi-Bio Science (SIMS), Soonchunhyang University, Cheonan 31151, Republic of Korea
[†] Co-first authors.
[‡] Co-corresponding authors.

Jaeseok Han;
Soonchunhyang Institute of Medi-bio Science (SIMS)
Soonchunhyang University, Cheonan, 31151
Republic of Korea
Tel: +82-41-413-5027; Fax: +82-41-413-5006; E-mail; hanjs015@sch.ac.kr

Hun Taeg Chung;
College of Korean Medicine,
Daegu Haany University, Gyeongsan, 38610,
Republic of Korea.
Tel: +83-53-819-1868; Fax: +82-53-819-1860; E-mail; chunght@dhu.ac.kr

Supplementary Figures

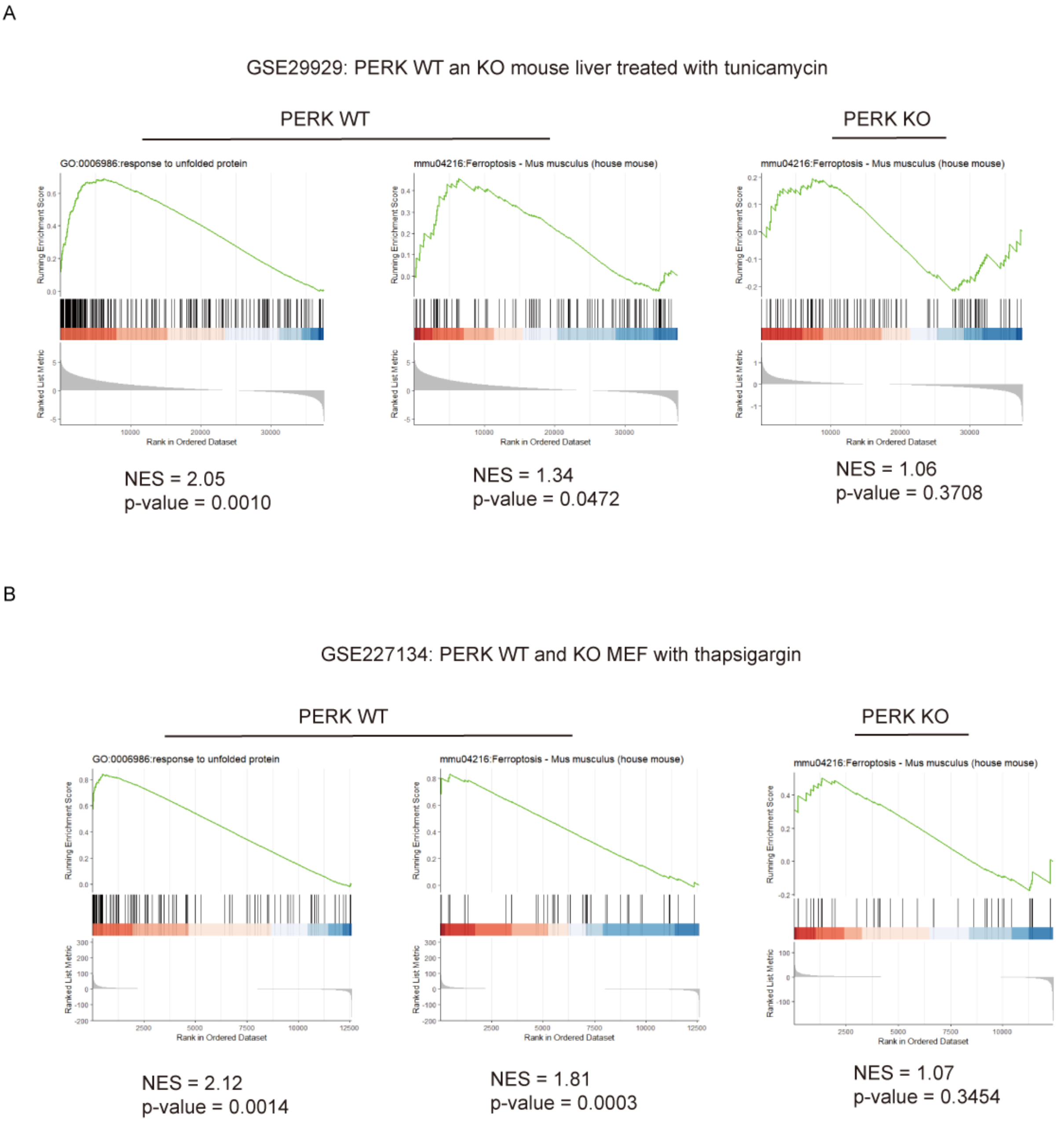

Supplementary Figure 1

Figure S1. Ferroptosis pathway is enriched under ER stress condition.

(A) GSEA plots of liver transcriptomes from C57BL/6 wide-type and liver-specific PERK

knockout (LsPERK-KO) mice injected with tunicamycin (1 mg/kg) for 6 hours. Data were obtained from GSE29929. **(B)** GSEA plots of PERK WT and PERK KO cells treated with thapsigargin (500 nM) for 3 hours. Data were obtained from GSE227134.

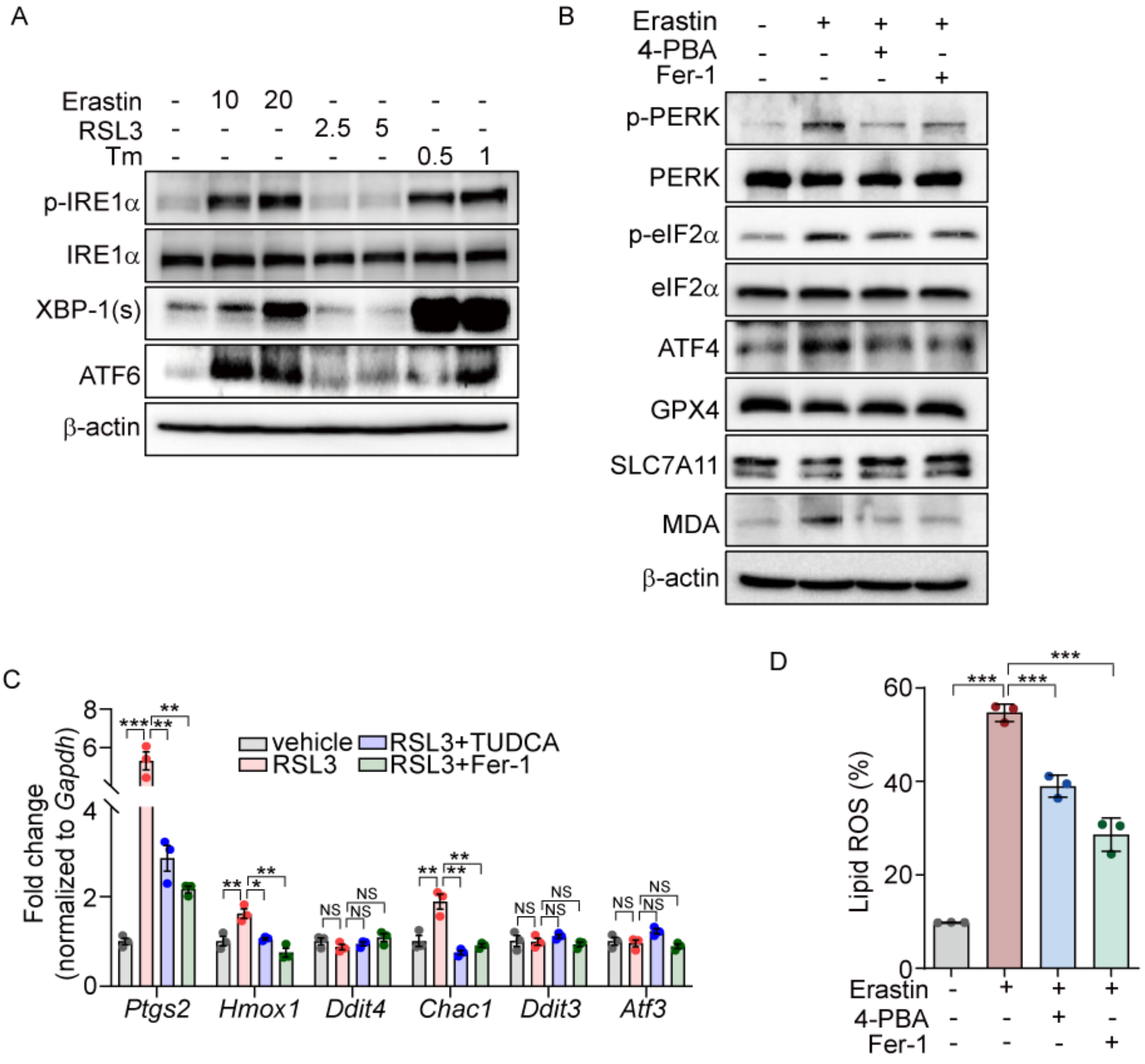

Supplementary Figure 2

Figure S2. ER chaperones inhibit erastin-mediated ferroptosis.

(A) Erastin, RSL3 and Tm were treated at indicated doses in AML12 cells. Western blot analysis of indicated proteins. **(B)** Western blot analysis of indicated proteins after erastin (10 μM) treatment in the presence or absence 4-PBA (0.5 mM) or Fer-1 (5 μM) for 24 hours. **(C)** Quantification of mRNA level of indicated genes after RSL3. **(D)** lipid ROS detected by BODIPY™ 581/591 C11 staining and analyzed by flow cytometry after erastin (10 μM) treatment in the presence or absence 4-PBA (0.5 mM) or Fer-1 (5 μM) for 24 hours. Data are mean ± SD (n=3); *$p<0.05$, **$p<0.01$* and ****$p<0.001$*.

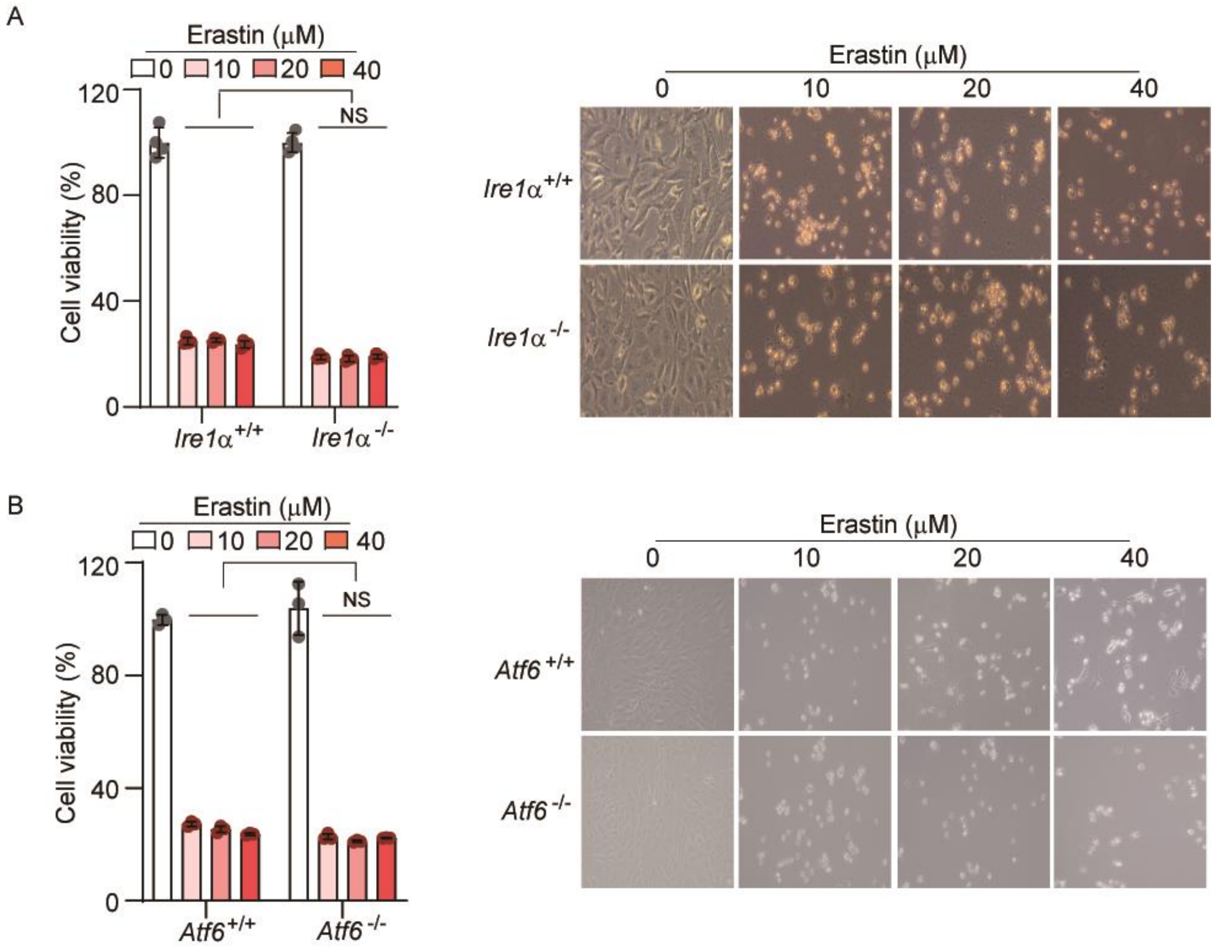

Supplementary Figure 3

Figure S3. Both IRE1α and ATF6 branches of UPR are not required for erstatin-mediated ferroptosis.

(A and B) Erastin was treated at indicated doses for 24 hours. (A) Cell viability assessed by MTT in *Ire1α*$^{+/+}$, *Ire1α*$^{-/-}$ hepatocyte cells (Left panel). Representative images of bright-field microscope with 10x magnification (Right panel). (B) Cell viability assessed by MTT in *Atf6*$^{+/+}$, or *Atf6*$^{-/-}$ hepatocyte cells (Left panel). Representative images of bright-field microscope with 10x magnification (Right panel). Data are mean ± SD (n=3); *$p<0.05$, **$p<0.01$ and ***$p<0.001$.

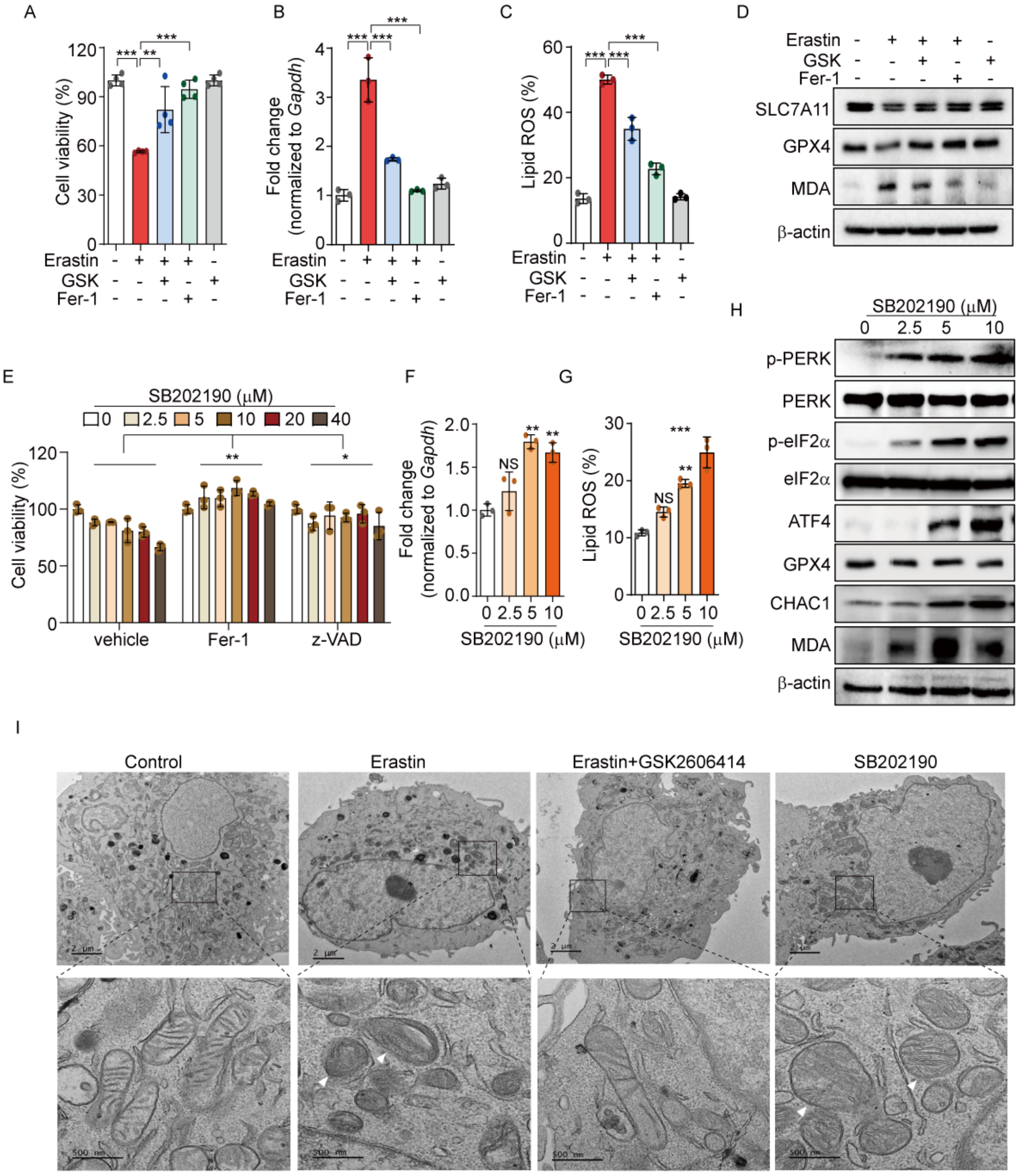

Supplementary Figure 4

Figure S4. PERK and its downsteam pathway is required for ferroptotic signaling activation.

(A-D) AML12 cells was treated with erastin (10 μM) in the presence or absence of GSK (0.5

μM) or Fer-1 (5 μM) for 24 hours **(A)** Cell viability assesed by MTT. **(B)** Quantification of mRNA level of *Ptgs2.* **(C)** lipid ROS detected by BODIPY™ 581/591 C11 staining and analyzed by flow cytometry. **(D)** Western blot analysis of indicated proteins. **(E-H)** SB202190 was treated at indicated doses for 24 hours in AML12 cells. **(E)** Cell viability assessed by MTT after Fer-1 (5 μM) or Z-VAD (20 μM) treatment. **(F)** Quantification of mRNA levels of *Ptgs2.* **(G)** lipid ROS detected by BODIPY™ 581/591 C11 staining and analyzed by flow cytometry. **(H)** Western blot analysis of indicated proteins. **(I)** Representative images of transmission electron microscopy. AML12 cells was treated with erastin (10 μM) in the presence or absence of GSK2606414 (0.5 μM) or SB202190 (20 μM) alone for 24 hours. White arrows Inindicate mitochondrial shrinkage, increased electron density, and rupture of the outer mitochondrial membrane. Data are mean ± SD (n=3); $^*p<0.05$, $^{**}p<0.01$ and $^{***}p<0.001$.

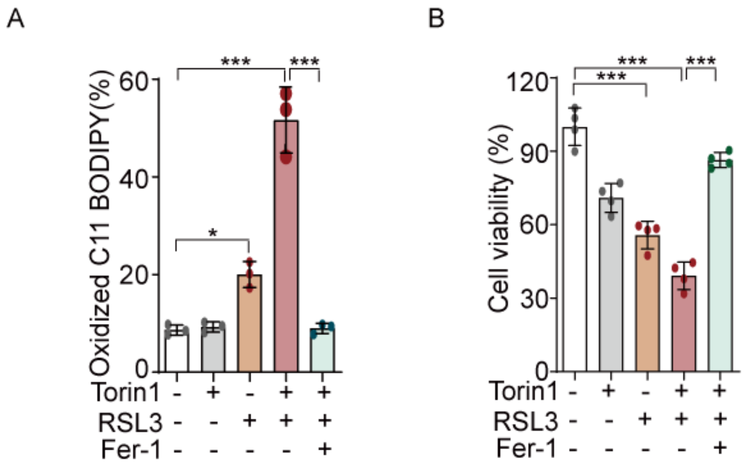

Supplementary Figure 5

Figure S5. Inhibition of mTORC1 by Torin1 sensitizes cells to RSL3-induced ferroptosis. (A and B) RSL3 (2.5 µM) or Torin1 (0.5 µM) were treated in the presence or absence of Fer-1 (5 µM) for 12 hours. **(A)** lipid ROS detected by BODIPY™ 581/591 C11 staining and analyzed by flow cytometry. **(B)** Cell viability assessed by MTT. Data are mean ± SD (n=3); *$p<0.05$, **$p<0.01$ and ***$p<0.001$.

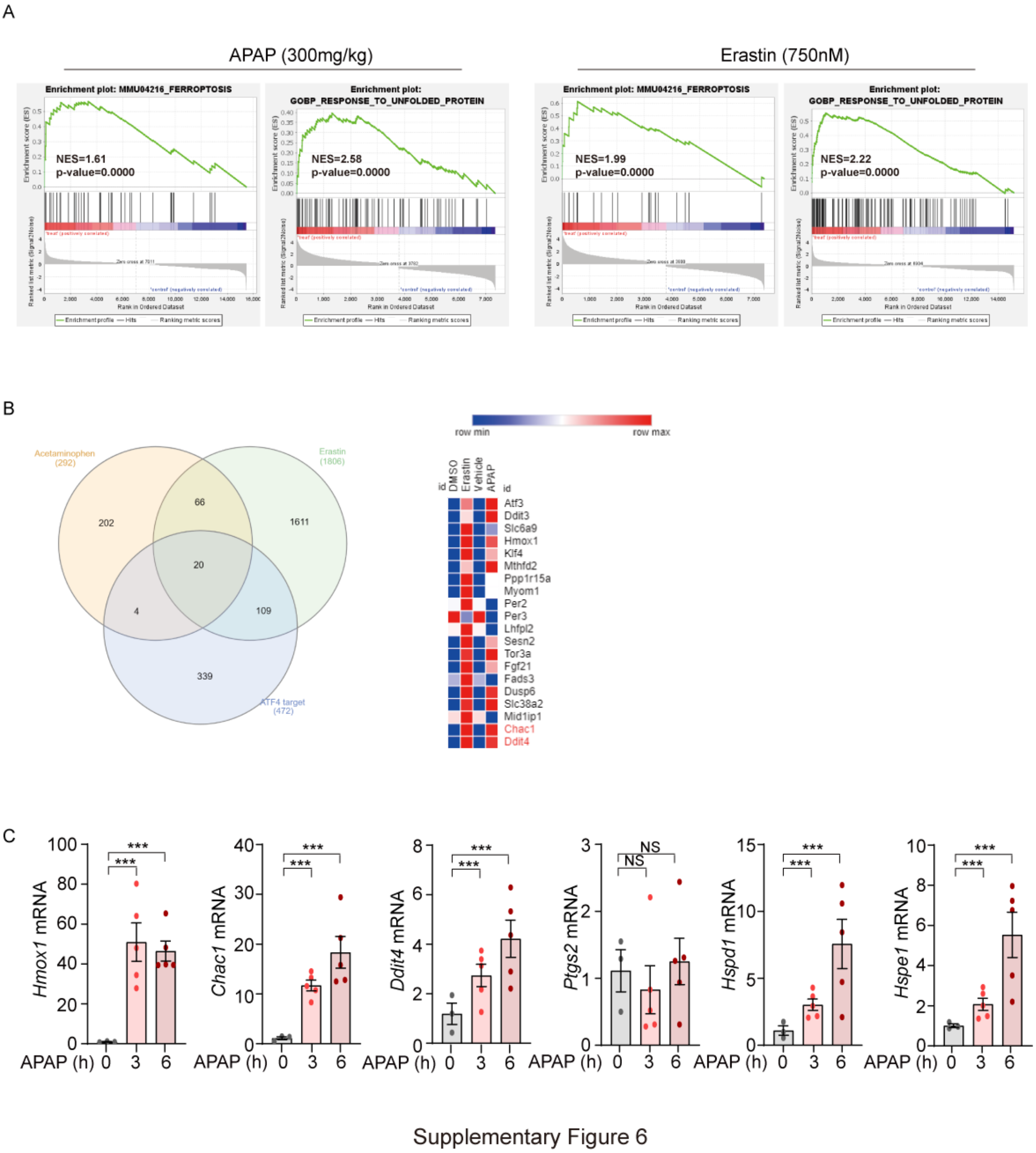

Figure S6. Ferroptosis pathway and response to unfolded protein is enriched under APAP and erastin treatment.

(A) GSEA plots of liver transcriptomes from C57BL/6 mice injected with APAP (300 mg/kg). Data were obtained from PRJNA744757 from NCBI-SRA. (B) GSEA plots of MEF transcriptomes treated with erastin (750nM) for 10 hours. Data were obtained from RNA-seq

performed in this study. (C) Intersection of upregulated genes (log2 fold change ≥ 1, p-value ≤ 0.05) from APAP treatment, erastin treatment, and ATF4's target genes. Heatmaps show the log2 fold change of these genes.
